# Identifying Genetic Players in Cell Sheet Morphogenesis Using a *Drosophila* Deficiency Screen for Genes on Chromosome 2R Involved in Dorsal Closure

**DOI:** 10.1534/g3.118.200233

**Published:** 2018-05-18

**Authors:** Richard D. Mortensen, Regan P. Moore, Stephanie M. Fogerson, Hellen Y. Chiou, Chimdindu V. Obinero, Neel K. Prabhu, Angela H. Wei, Janice M. Crawford, Daniel P. Kiehart

**Affiliations:** Biology Department, Duke University, Durham, NC, 27708, USA

**Keywords:** amnioserosa, lateral epidermis, actomyosin, morphogenesis, dorsal closure

## Abstract

Cell sheet morphogenesis characterizes key developmental transitions and homeostasis, in vertebrates and throughout phylogeny, including gastrulation, neural tube formation and wound healing. Dorsal closure, a process during *Drosophila* embryogenesis, has emerged as a model for cell sheet morphogenesis. ∼140 genes are currently known to affect dorsal closure and new genes are identified each year. Many of these genes were identified in screens that resulted in arrested development. Dorsal closure is remarkably robust and many questions regarding the molecular mechanisms involved in this complex biological process remain. Thus, it is important to identify all genes that contribute to the kinematics and dynamics of closure. Here, we used a set of large deletions (deficiencies), which collectively remove 98.5% of the genes on the right arm of *Drosophila melanogaster’s* 2^nd^ chromosome to identify “dorsal closure deficiencies”. Through two crosses, we unambiguously identified embryos homozygous for each deficiency and time-lapse imaged them for the duration of closure. Images were analyzed for defects in cell shapes and tissue movements. Embryos homozygous for 47 deficiencies have notable, diverse defects in closure, demonstrating that a number of discrete processes comprise closure and are susceptible to mutational disruption. Further analysis of these deficiencies will lead to the identification of at least 30 novel “dorsal closure genes”. We expect that many of these novel genes will identify links to pathways and structures already known to coordinate various aspects of closure. We also expect to identify new processes and pathways that contribute to closure.

Biological form and structure are generated through a multi-step process that requires cascades of gene expression and changes in signaling pathways to orchestrate cell fate determination and cell differentiation (pattern formation), which are a prelude to the cell shape changes and rearrangements that constitute morphogenesis – such movements transform cellular sheets into the complex structures required for both embryonic and adult function. Morphogenesis is essential to development in all multicellular organisms and requires the coordination of signaling pathways that regulate cell structures, including the cytoskeleton, and adhesion to perform cell and tissue movements. The dorsal closure stage of embryogenesis in *Drosophila melanogaster* is a genetically tractable model system in which to study epithelial cell sheet morphogenesis and is comparable to vertebrate morphogenic movements that involve epithelial fusion such as gastrulation, heart morphogenesis, neural tube closure and palate formation (Stalsberg and Dehaan 1969; Hashimoto *et al.* 1991; Pai *et al.* 2012; Ray and Niswander 2012; Heisenberg and Bellaiche 2013; Kim *et al.* 2015). Many of the genes and mechanisms involved in dorsal closure are conserved across phylogeny and also share salient features with wound healing processes (Harden 2002; Heisenberg 2009; Belacortu and Paricio 2011; Ray and Niswander 2012; Heisenberg and Bellaiche 2013; Razzell *et al.* 2014; Hashimoto *et al.* 2015; Begnaud *et al.* 2016; Gorfinkiel 2016; Hayes and Solon 2017; Kiehart *et al.* 2017).

Dorsal closure is a 3-4 hr developmental process during mid-embryogenesis whereby lateral epidermal sheets from either side of the embryo elongate toward the dorsal midline where they meet and fuse to form a seamless epithelium (reviewed most recently in Hayes and Solon 2017; Kiehart *et al.* 2017). At the onset of closure, the dorsal surface between the two-advancing lateral epidermal sheets is filled by a thin, squamous epithelium called the amnioserosa (AS; Figure 1A). The amnioserosa cells are isodiametric in shape (Schöck and Perrimon 2002; Pope and Harris 2008; Lynch *et al.* 2013) with actomyosin-rich, apical junctional belts and medioapical arrays that contribute to their contractility as the cells oscillate or pulsate and provide force(s) for closure (Fernández *et al.* 2007; Blanchard *et al.* 2009; Solon *et al.* 2009; Blanchard *et al.* 2010; David *et al.* 2010; Sokolow *et al.* 2012; Wells *et al.* 2014; Gorfinkiel 2016; R. P. Moore, U. S. Tulu, L. Dong, W. R. Legant, A. H. Cox, *et al.*, unpublished data). As dorsal closure progresses, the amnioserosa cells thicken radially, shorten along the circumference of the embryo perpendicular to the anterior-posterior axis and ingress from the tissue surface where they undergo apoptosis (Kiehart *et al.* 2000; Narasimha and Brown 2004; Reed *et al.* 2004; Toyama *et al.* 2008; Lennox and Stronach 2010; Muliyil *et al.* 2011; Sokolow *et al.* 2012; Shen *et al.* 2013; Beira *et al.* 2014; Muliyil and Narasimha 2014; Saias *et al.* 2015). Early in closure, actin and myosin are recruited to the leading edge of the dorsal-most cells of the lateral epidermis (termed DME cells, Figure 1A) forming a contractile purse string and providing another force for closure (Young *et al.* 1993; Hutson *et al.* 2003; Franke *et al.* 2005; Peralta *et al.* 2007). The DME cells form an integrin-dependent interface with the peripheral-most amnioserosa cells (PAS cells, Figure 1B”; see also Figure 1 in Rodriguez-Diaz *et al.* 2008) in which the DME and PAS cells become reciprocally wedge-shaped during closure thereby increasing the shared surface area that is also joined by adherens junctions (Kaltschmidt and Brand 2002; Narasimha and Brown 2004; Kiehart *et al.* 2017). At the anterior and posterior ends of the dorsal opening, the two sheets of lateral epidermis meet to form canthi and give the dorsal opening an eye shape with characteristic curvature of the purse strings (Figure 1B”; Hutson *et al.* 2003). As closure progresses, the two sheets zip together at both canthi, aligning patterned tissue segments and providing additional forces that coordinate changes in the width (along the anterior-posterior axis) and the height (along the dorsal-ventral axis) of the dorsal opening and are essential for the end stages of closure. Zipping is mediated by interdigitation of actin-rich filopodia and the overlap of microtubule-rich lamellar sheets to form a seamed, and later a seamless epithelium (Jacinto *et al.* 2000; Hutson *et al.* 2003; Gates *et al.* 2007; Wada *et al.* 2007; Millard and Martin 2008; Eltsov *et al.* 2015; Lu *et al.* 2015).

**Figure 1:**
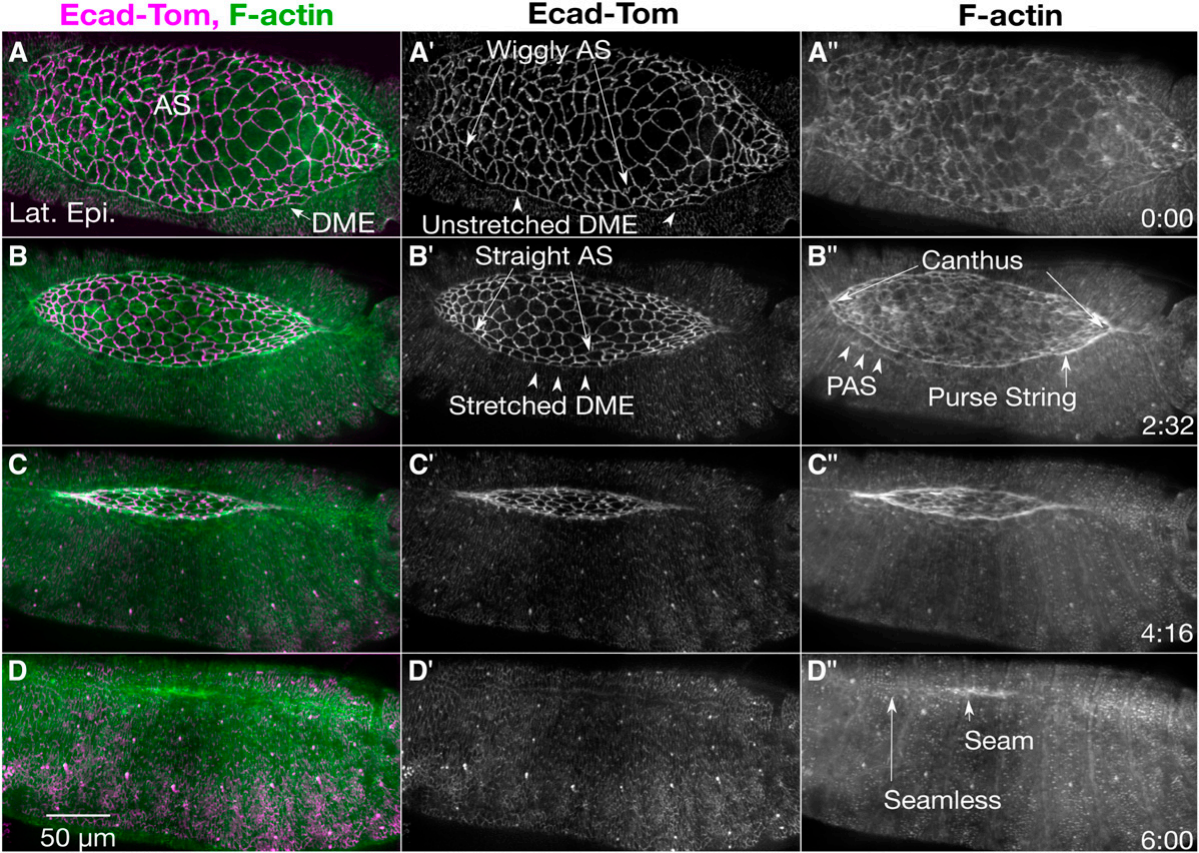
Time-lapse image series of dorsal closure from pre-canthus formation to a seamed epithelium. The dynamic changes in cellular morphologies and the cytoskeleton during dorsal closure are observed by labeling the cadherin junctions (Ecad-Tomato, A’-D’) and F-actin (sGMCA, a GFP tagged actin-binding domain of moesin, A”-D”), respectively. Prior to dorsal closure, the anterior end of the dorsal opening is blunt and the posterior end is rounded, the boundary between the dorsal most epithelia (DME) and peripheral amnioserosa (PAS) is scalloped (A-A”), the amnioserosa (AS) cell boundaries are wiggly (A’), and actin is weakly localized to the purse strings (A”). At the onset of dorsal closure, canthi form at the anterior and posterior ends of the dorsal hole (B’’), the amnioserosa cell borders straighten out (B’), actin accumulates at the DME/PAS boundary forming the supracellular actomyosin purse strings (B”), and the PAS cells tuck under the DME (B-B”). As dorsal closure progresses, the dorsal opening decreases in height and width (C-C”). At the conclusion of dorsal closure, there is a seamed, and eventually seamless, epithelium (D-D”). Anterior is to the left, posterior to the right in all confocal micrographs. Time is in hr:min. The scale bar in D applies to all micrographs (50 µm).

Multiple signaling pathways and cellular components regulate or participate in the process of dorsal closure. Small GTPases in the Rho superfamily, non-receptor tyrosine kinases, the Wg/Wnt pathway, the Notch pathway, the JNK pathway, the BMP/Dpp pathway and the insect steroid hormone ecdysone are all key regulators of dorsal closure (Harden 2002; Gilbert 2004; Woolner *et al.* 2005; Vanhook and Letsou 2008; Harris *et al.* 2009; Belacortu and Paricio 2011; Gorfinkiel and Blanchard 2011; Munoz-Soriano *et al.* 2012; Ríos-Barrera and Riesgo-Escovar 2013). It is interesting to speculate that one of these signaling cascades might trigger the onset of closure, possibly JNK which is localized at the interface between the lateral epidermis and amnioserosa where it promotes the formation of the actomyosin purse string and is upregulated when one of the tissues is compromised (reviewed in Kiehart *et al.* 2017). These upstream signaling cascades orchestrate the dorsal closure process by regulating downstream cellular components, *e.g.*, regulation of the actomyosin cytoskeleton, which when linked to junctional complexes provides structural integrity and mechanical properties to alter cell shapes and the contractile forces of morphogenesis (Kiehart *et al.* 2000; Franke *et al.* 2005; Gorfinkiel and Martinez-Arias 2007; Harris 2012; Jayasinghe *et al.* 2013). Similar regulation of the microtubule cytoskeleton plays a key role in the zipping process, the final step of closure that results in fusion of the two lateral epidermal sheets (Jankovics and Brunner 2006; Fernández *et al.* 2007; Almeida *et al.* 2011; Eltsov *et al.* 2015; Takács *et al.* 2017).

The complexity of dorsal closure requires an understanding of the molecular detail that dictates how cells are specified to contribute to closure, how the molecular and cellular components that drive closure are assembled in these cells, how the cells undergo choreographed cell shape changes and movements, and how these changes and movements result in morphogenesis. Dorsal closure exhibits emergent properties and is a robust, resilient and redundant developmental process. Closure is robust as demonstrated by evidence that when force from one tissue is removed either genetically or through laser surgery, other tissues are able to manage closure, often at native rates (Hutson *et al.* 2003; Franke *et al.* 2005; Peralta *et al.* 2007; Wells *et al.* 2014; Pasakarnis *et al.* 2016; Kiehart *et al.* 2017). For example, when apoptosis is inhibited in the amnioserosa, closure still completes (Toyama *et al.* 2008; Muliyil and Narasimha 2014). When myosin function is compromised in either the purse strings or the amnioserosa cells, closure still completes (Franke *et al.* 2005; Duque and Gorfinkiel 2016; Pasakarnis *et al.* 2016) – if myosin function is compromised in both tissues, closure fails. Closure is resilient in that it can complete even when the tissues involved are abnormally shaped and lack cytoskeletal components as in *wingless* mutant embryos (Mcewen and Peifer 2000; Kaltschmidt and Brand 2002; Morel and Arias 2004). And closure is redundant in that similar protein products can be encoded by multiple genes as is the case with Src and Rac signaling pathways (Tateno *et al.* 2000; Woolner *et al.* 2005). To understand in detail the process of dorsal closure, it is ultimately important to identify all the gene products that contribute to closure.

Genetic screens have provided a valuable source for identifying the genes involved in various biological processes. Systematic mutant screens to find various genes that encode components of biochemical pathways were performed in bacteria and fungi (Beadle and Tatum 1941) and similar screens in bacteriophage identified the pathways to phage assembly (Wood *et al.* 1968; Wood 1973). Mutant screens in yeast were also integral in identifying key components of the cell cycle (Hartwell *et al.* 1970; Nurse *et al.* 1976). While classical genetic mutant screens in *Drosophila melanogaster* identified many of the genes involved in pattern formation and morphogenesis during embryonic development (Nüsslein-Volhard and Wieschaus 1980; Jürgens *et al.* 1984; Nüsslein-Volhard *et al.* 1984; Wieschaus *et al.* 1984), these screens relied on the presence of a dorsal hole in the larval cuticle to identify genes involved in dorsal closure. Later screens were performed to knock down maternally loaded candidate genes that are deposited in the egg, again assaying the presence of a dorsal hole in the larval cuticle (Schüpbach and Wieschaus 1986b; Schupbach and Wieschaus 1986a; Nusslein-Volhard *et al.* 1987; Schupbach and Wieschaus 1989). By assessing a post-embryogenesis phenotype, only mutations in genes that lead to the failure of dorsal closure are identified. Because dorsal closure is a robust and resilient process, many mutants are able to complete closure even when the tissues and processes that contribute to normal closure are severely disrupted. Thus, although these classical screens identified a substantial fraction of the ∼140 genes involved in dorsal closure, many other genes that contribute to dorsal closure were not identified due to the inability to study in real-time the kinematics and dynamics of dorsal closure.

A more recent screen for dorsal closure genes used *in vivo* time-lapse video microscopy, and an RNAi-based, loss-of-function screen to analyze the role of candidate genes during closure. Six novel dorsal closure genes with phenotypes more subtle than a catastrophic failure of closure were identified (Jankovics *et al.* 2011) with biological functions that include pattern formation, signal transduction, vesicle trafficking and cytoskeletal regulation. Although this RNAi-based candidate gene screen failed to identify some genes included in the candidate pool that were previously known to affect dorsal closure, the identification of new dorsal closure genes offers strong evidence for the need of a more complete screen of the genome using direct time-lapse imaging of dorsal closure stage embryos.

To gain a better understanding of morphogenesis in the process of dorsal closure and homologous morphogenic movements in development and wound healing, it is important to generate a more complete understanding of *all* of the genes whose zygotic expression is essential for normal closure. Systematic removal of genomic regions in combination with time-lapse fluorescence microscopy provides an ideal combination for a screen of zygotically expressed genes involved in dorsal closure. Fluorescence time-lapse imaging identifies more subtle defects that were missed by previous classical screens (Nüsslein-Volhard and Wieschaus 1980; Jürgens *et al.* 1984; Nüsslein-Volhard *et al.* 1984; Wieschaus *et al.* 1984; Schüpbach and Wieschaus 1986b; Schupbach and Wieschaus 1986a; Nusslein-Volhard *et al.* 1987; Schupbach and Wieschaus 1989). Arguably, the mutants that disrupt dorsal closure, but still complete closure are just as valuable to our understanding of the process of dorsal closure as those that fail, as they help us understand the cell and tissue behaviors underlying the robust nature of this process.

The Bloomington *Drosophila* Stock Center deficiency kit consists of 473 deficiencies that collectively delete 98.3% of annotated genes (Cook *et al.* 2012). Regions not covered by the kit are mostly due to haplolethal or haplosterile regions. Most of the deficiencies are molecularly defined and their breakpoints are mapped to single bases, defining all genes completely or partially deleted in each deficiency. The kit is further divided into six groups covering the X chromosome and each of the autosomal chromosomal arms (1, 2L, 2R, 3L, 3R, 4). There are no deficiencies for the Y chromosome in the kit; however, the removal of the Y chromosome is not lethal and XO males are morphologically indistinguishable from XY males except that they are sterile. This suggests that the Y chromosome has no genes that contribute in any substantial way to dorsal closure. Yet, given the robust nature of dorsal closure, it is possible the Y chromosome contains genes that contribute to dorsal closure in subtle ways that could be identified by fluorescent time-lapse imaging. Although each of the deficiencies in this kit removes a large number of genes, the use of additional available overlapping deficiencies narrows the interval of breakpoints to a median of just nine genes (Cook *et al.* 2012). With the use of genomic duplications and available mutants in the deleted region of interest, and in conjunction with tools such as CRISPR to create sub-Dfs or knockout individual genes, it is feasible to efficiently narrow deficiency regions down to individual genes. The use of the Bloomington deficiency kit gives almost complete coverage of the genome and is ideal for near-saturation in a forward genetics screen.

Here we describe the use of the Bloomington *Drosophila* deficiency (Df) kit to perform a pilot screen for zygotic mutations that affect dorsal closure on the right arm of the second chromosome (2R). Two crosses allow us to homozygose each Df in a genetic background that ubiquitously expresses E-cadherin-GFP (Ecad-GFP). We can then image these homozygous embryos, observing *in vivo* the cell and tissue movements throughout closure. We screened for defects in a variety of tissue movements that contribute to dorsal closure and have identified “dorsal closure Dfs” that when homozygosed, cause defects in closure. Such defects range from strong phenotypes resulting in the failure of closure, strong phenotypes in which closure still completes but one (or more) tissue movement is perturbed, to weak phenotypes in which closure completes with defects that only slightly diverge from closure in wild type, control embryos. Of the 92 Dfs available for 2R, embryos homozygous for 88 Dfs were successfully imaged. Of these, 47 are “dorsal closure Dfs”, *i.e.*, they cause a demonstrable, dorsal closure phenotype. Eighteen of these Dfs do not delete any previously identified genes that contribute to dorsal closure (*i.e.*, they delete no known “dorsal closure gene or genes”). Further characterization of a small subset of these 47 Dfs yielded the identification of four new dorsal closure genes to date. Eleven of the 47 “dorsal closure Dfs” cause pre-closure defects some of which become more severe as dorsal closure progresses while others do not. Although these Dfs may or may not delete genes that contribute directly to the process of closure during dorsal closure, the aberrant cell and tissue morphologies can be instructive and provide valuable insight into the robustness of closure. Given the diversity of the phenotypes we identified that affect all tissues and processes that contribute to dorsal closure, this near-saturation pilot screen demonstrates that a number of discrete processes comprise dorsal closure and are susceptible to mutational disruption. By extension, a whole-genome, forward genetics Df screen will provide new insight into the multiple molecular mechanisms that contribute to cell sheet morphogenesis. Extrapolating from the 3009 genes on 2R that we have assessed in this pilot screen, we anticipate that a full genome Df screen would identify ∼165 or more new dorsal closure genes, more than doubling the number of genes currently known to be involved in dorsal closure. As these genes are identified and we have a more complete understanding of the processes and the genes that contribute to closure, we can begin to develop a more mature understanding of the molecular mechanisms behind the evolutionarily conserved processes of cell sheet morphogenesis.

## Materials And Methods

### Drosophila stocks

The 2R Deficiency (Df) Kit stocks were obtained from the Bloomington Stock Center (Bloomington, IN). For easier reference in the screen, Dfs were numbered based on the chromosomal position of their proximal breakpoints, from Df(2R)1 which is centromere proximal, to Df(2R)92 which is centromere distal. The genetic names of each Df are listed with their Bloomington stock number and the number that we assigned in Supplemental Appendix A. Additionally, any overlapping Dfs used are listed in Supplemental Appendix B. The CyO, twist-Gal4 balancer is from the *wg^Gla-1^*/CyO, twist-Gal4::UAS-2xEGFP stock, BSC #6662 (Halfon *et al.* 2002). Control refers to fly stocks that were *w*^-^ and ubiquitously express one of two transgenes: sGMCA (to label F-actin) or Ecad-GFP (to label cell-cell junctions, Kiehart *et al.* 2000; Oda and Tsukita 2001). All other stocks used are derived from stocks also available from the Bloomington *Drosophila* Stock Center unless otherwise noted.

### Crosses

**Figure 2:**
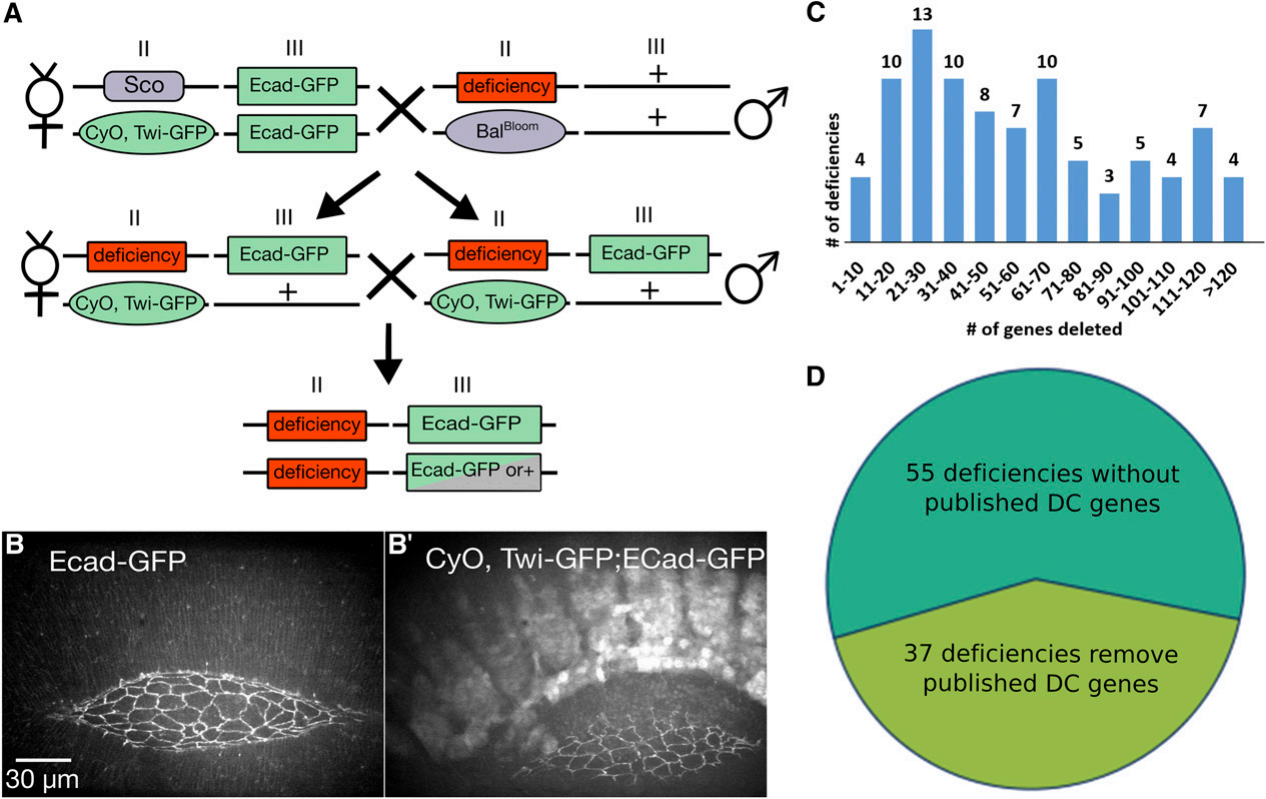
Deficiency screen crossing scheme and the 2R Df kit. Embryos homozygous for the Df are collected after two crosses (A). Bal^Bloom^ is the balancer supplied with the Df stock from the Bloomington Stock Center. Roman numerals indicate 2^nd^ (II) or 3^rd^ (III) chromosomes. Homozygous Df embryos are selected by the lack of Twi-GFP on the balancer chromosome (B-B’). 2R Dfs remove from three to more than 120 genes (C). Of the 92 Dfs on the 2R, 37 remove genes known to affect dorsal closure as documented in the literature (D). The scale bar in B also applies to B’ (30 µm).

All crosses were performed at 25° on standard cornmeal/molasses fly food. We were able to evaluate the effect each Df has on dorsal closure through two crosses (Figure 2). The first cross balanced the Df of interest over a marked balancer with an “imaging” stock of Ecad-GFP (or sGMCA, a GFP tagged actin-binding domain of moesin) on the third chromosome: male flies of the genotype Df(2R)n/Bal^Bloomington^ (abbreviated Bal^Bloom^) were crossed to virgin females of the genotype *sna^Sco^*/CyO, twist-Gal4::UAS-2xEGFP (TGC); Ecad-GFP (Figure 2A). The second cross was an *inter se* cross of 7-10 males and 15-20 virgin female progeny of the genotype Df(2R)n/TGC; Ecad-GFP/+ and embryos were collected from this cross for imaging (see below). We constructed the “imaging” stock *sna^Sco^*/TGC; Ecad-GFP for the screen. We chose Ecad-GFP for our screen because its fluorescence comes from a ubiquitously expressed E-cadherin-GFP transgene that labels junctional belts (Oda and Tsukita 2001), making it ideal for the analysis of cell shapes (assessed in two dimensions near the apical ends of the cells). Moreover, while all transgenes have the potential for introducing artifact, in our experience, artifacts due to this transgene are minimal and relate to the rescue of the Dfs that cover the cadherin locus or other loci involved in cadherin-based adhesion (see Discussion).

### Imaging

Embryos collected from 2-4 h egg lays were aged ∼24 h at 16° until late germband retraction or early dorsal closure stages. Embryos were manually sorted using a Zeiss Discovery V12 SteREO dissecting microscope (Carl Zeiss, Thornwood, NY) equipped to detect green fluorescence to select Df(2R)n/Df(2R)n; Ecad-GFP/Ecad-GFP or /+. Such embryos are easily distinguished from siblings that carry the fluorescent balancer TGC (Figure 2B). In addition, we imaged *sna^Sco^*/+; Ecad-GFP/+ or +/+; Ecad-GFP as controls with each Df.

Embryos were prepared for imaging as previously described (Kam *et al.* 1991; Kiehart *et al.* 1994). Of the 10-12 mounted embryos for each genotype, 3-4 dorsal side up embryos in early to mid-dorsal closure were selected for “4D” imaging (x, y, z and t). To view the whole dorsal opening and due to the curvature of the embryo, we used either a Zeiss multi-immersion 40X, 0.9 N.A. objective, a Zeiss oil-immersion 40X, 1.3 N.A. objective or a Zeiss water-immersion 40x, 1.2 N.A. objective to image multiple z planes for each embryo at each time point. Images were acquired on a Zeiss Axiovert 200M or a Zeiss Axio Imager.M2m confocal using Metamorph software (Molecular Devices, San Jose, CA) or Micro-Manager software (Open Imaging, San Francisco, CA). We typically imaged 16 z-planes, 1μm apart, to be able to view the full dorsal opening in early dorsal closure. Each z-series for each embryo was imaged every two minutes until all embryos completed closure or closure failed. We imaged a minimum of six embryos of each Df over 2-3 imaging sessions. All embryos were imaged at room temperature (23-25°) and fluctuations in daily room temperature were minor.

### Analysis

Fiji/ImageJ2 (NIH, Schneider *et al.* 2012) was used to compile maximum intensity projection movies for review of each embryo in each experimental run. We used the movies to visually (qualitatively) assess the morphology and dynamics of the amnioserosa and lateral epidermal cells and classified the phenotype of each homozygous Df according to its severity and which tissues were affected (see below). The analyst assessed dorsal closure blindly during this process (*i.e.*, s/he did not know whether the Df removed a published dorsal closure gene), allowing for an unbiased classification of the Df dorsal closure phenotype. Once all Dfs had been analyzed, the phenotypes of Dfs that removed known dorsal closure genes were compared to the published phenotype for the dorsal closure gene to determine whether the dorsal closure Df phenotype could be fully explained by the known gene.

### Image processing

Images were processed using Fiji/ImageJ2 (NIH, Schneider *et al.* 2012). The deepest z planes pick up auto fluorescence from the underlying yolk making it difficult to view the cell shapes of the amnioserosa and lateral epidermis. For figure quality images, the yolk was masked following the mask projection method described previously (Sokolow *et al.* 2012, see their Figure S1). Image stacks were reduced to a single plane using a maximum intensity projection protocol. We improved fluorescence signal to noise with background subtraction using a rolling ball radius of 50-200 pixels, with the sliding paraboloid and smoothing features both disabled. To better define cell shapes, images were further processed using the unsharp mask filter with a radius of 1 pixel and mask weight of 0.30.

### Statistical analysis

All statistical analysis was performed using Graphpad Prism 7.04. A 1-way ANOVA was used to determine if there were differences between multiple genotypes. Upon finding a significant difference, a Tukey’s multiple comparison test was performed to determine which genotypes were different. We report the multiplicity adjusted P-values. The data sets to test the rate of closure had equal variances, therefore we used an unpaired T-test to determine if there were significant differences between the two genotypes. Because the variances in ingression rates were unequal, we used a Welch’s test to determine if there were differences between the two genotypes. P-values less than 0.05 are reported.

### Data Availability

Reagents are available upon request. The authors state that all data necessary for confirming the conclusions presented in the article are represented fully within the article. Supplemental material is available at Figshare: https://doi.org/10.25387/g3.6207470.

## Results & Discussion

### The screen

Two crosses introduced into each 2R Df kit stock a marked balancer chromosome and one or two copies of E-cadherin-GFP (Ecad-GFP), an “imaging” transgene located on the third chromosome (Figure 2A). Homozygous Df embryos were identified by the absence of the balancer chromosome, which was marked by a bright GFP signal expressed in mesoderm (Figure 2B’). The ubiquitously expressed Ecad-GFP transgene labels junctional belts (Oda and Tsukita 2001), making it ideal for the analysis of cell shapes. Moreover, while all transgenes have the potential for introducing artifact, in our experience, artifacts due to this transgene are usually minimal or non-existent (see below). The 92 Dfs in the 2R Df kit remove 3043 annotated genes in total, comprising 98.5% of 2R euchromatin: each Df deletes 3-216 genes with a median of 50.5 genes removed per Df (Figure 2C). Previously identified genes involved in dorsal closure are present in 37 of the 92 Dfs (Figure 2D). Of the 92 Dfs available for the 2R, 88 were successfully imaged during closure (see below). Dfs were imaged “blindly”, *i.e.*, whether or not a given Df deleted a known dorsal closure gene was not known when it was imaged. We analyzed movies generated from *in vivo* time-lapsed images of all 88 Dfs to assess defects in dorsal closure based on the following criteria: Are the shapes of the amnioserosa cells and lateral epidermal cells comparable to those in control embryos? Do the shapes of the amnioserosa and lateral epidermal cells change during closure comparably to those seen in control embryos? Are the canthi well formed? Does the initially scalloped leading edge of the lateral epidermis resolve into a smooth arc as closure progresses? Does the embryo complete closure in a timely fashion? Movies generated from time-lapse records for six or more embryos were independently scored for each of the questions posed to visually (qualitatively) assess the phenotype of each homozygous Df according to its severity and which tissues were affected (see below). Of the 88 Df stocks imaged, 47 Dfs cause a substantial phenotype and 18 of these Dfs have no previously known dorsal closure genes in the deleted region.

We sorted the Dfs into five categories based on the severity of the observed dorsal closure phenotype in comparison to controls (see Appendix A). Thirteen Dfs cause a phenotype classified as “strong and fails”. These embryos have severe defects in one or more of the processes that contribute to dorsal closure and result in a failure of closure in some or all of the embryos analyzed. Fourteen Dfs cause a “strong but still closes” phenotype. In this group, the phenotype is also severe with atypical cell shapes and/or behaviors, but surprisingly, all embryos still complete closure although often with noticeable defects in the formed, dorsal epithelium (*e.g.*, scarring or puckering), which results from closure. Twenty Dfs are classified as “mid-severity” and cause a penetrance of over 50% that is less severe than strong phenotypes but are clearly distinguishable from control embryos. Ten Dfs are classified as “weak” and cause a less penetrant phenotype (under 50%) and are less easily distinguished from control embryos. Finally, 31 Dfs are classified as “no phenotype” and are essentially indistinguishable from controls. (Note that in all categories, some of these homozygous Dfs cause phenotypes that are not highly penetrant and some cause multiple phenotypes some of which are not particularly penetrant. This is potentially due to the maternal load of gene products that perdure until the time of dorsal closure.) Here we focus on the 47 Dfs that cause phenotypes categorized as “strong and fails”, “strong but still closes” and “mid-severity”.

The phenotypes displayed by embryos homozygous for several of the Dfs have defects in multiple tissues (summarized in Appendix A), and best efforts were made to group Dfs by the tissue or process affected: the amnioserosa, the lateral epidermis, the zipping process, canthus structure, and/or the interface between the lateral epidermis and the amnioserosa (Table 1, Table 2, Table 3, and Table 4, respectively). Phenotypes are classified as affecting the amnioserosa if they have irregular amnioserosa cell shapes, the amnioserosa falls apart, or there are abnormal amnioserosa ingressions (Table 1). Lateral epidermis phenotypes include large cell areas, isotropically shaped cells that are not elongated circumferentially, or disorganized lateral epidermal cells (not organized in regular, elongated rows, Table 2). Dfs are classified as causing zipping/canthus phenotypes if the epidermis is puckered or scarred after closure, the dorsal opening becomes long and skinny (cigar-shaped), possibly due to hindered zipping, or a canthus or canthi is (are) missing or malformed (Table 3). Several Dfs affect the interface between the amnioserosa and lateral epidermis with phenotypes including a wavy or round dorsal opening or tearing along the border between the amnioserosa and leading edge of the lateral epidermis (Table 4).

**Table 1:** Deficiencies with amnioserosa phenotypes (20 total)

Amnioserosa Phenotype	Number of Dfs	Screen Name Df(2R)n
Irregular amnioserosa cell shapes	**16**	03, 04, 06, 08, 09, 12, 13, 14, 28, 32, 33, 40*, 47, 71, 84, 90
Amnioserosa falls apart	**6**	22, 40*, 75, 83, 85, 90
Abnormal amnioserosa ingressions	**3**	22, 28, 84

Deficiencies are separated into three amnioserosa groups: irregular amnioserosa cell shapes, amnioserosa falls apart, and abnormal amnioserosa ingressions. The asterisk indicates that Df(2R)40 has a severe dorsal closure phenotype because of a lesion that falls outside of the mapped Df (see text). In this table, we refer to the Dfs by the screen name; the corresponding Bloomington stock number can be found in Appendix A.

**Table 2:** Deficiencies with lateral epidermis phenotypes (15 total)

Lateral Epidermis Phenotype	Number of Dfs	Screen Name Df(2R)n
Large cell areas	**6**	16, 17, 35, 37, 60, 61
Isotropic/non-stretched cells	**4**	11, 16, 17, 91
Disorganized cells	**8**	08, 09, 22, 32, 40*, 44, 61, 62

Deficiencies are separated into three lateral epidermis phenotype groups: large lateral epidermal cell areas, isotropic or non-stretched lateral epidermal cells, and disorganized lateral epidermal cells. The asterisk indicates that Df(2R)40 has a severe dorsal closure phenotype because of a lesion that falls outside of the mapped Df (see text). In this table, we refer to the Dfs by the screen name; the corresponding Bloomington stock number can be found in Appendix A.

**Table 3:** Deficiencies with zipping/canthus phenotypes (32 total)

Zipping/Canthus Phenotype	Number of Dfs	Screen Name Df(2R)n
Scarring from zipping	**14**	03, 04, 07, 09, 12, 22, 28, 35, 37, 47, 60, 61, 71, 90
Cigar shaped opening	**11**	02, 04, 05, 06, 07, 08, 12, 45, 46, 64, 71
Missing/malformed canthus	**13**	07, 09, 10, 11, 16, 17, 24, 32, 33, 62, 72, 91, 92

Deficiencies are separated into three zipping/canthus phenotypes: scarring from zipping, cigar shaped dorsal opening, and missing or malformed canthus/canthi. In this table, we refer to the Dfs by the screen name; the corresponding Bloomington stock number can be found in Appendix A.

**Table 4:** Deficiencies with phenotypes at the interface between the amnioserosa and lateral epidermis (20 total)

Phenotype at Interface Between amnioserosa and lateral epidermis	Number of Dfs	Screen Name Df(2R)n
Wavy dorsal opening	**9**	04, 10, 11, 18, 22, 45, 46, 66, 84
Round dorsal opening	**4**	03, 23, 33, 50
Tearing along the amnioserosa/lateral epidermis border	**9**	08, 09, 11, 17, 18, 20, 21, 40*, 63

Deficiencies are separated into three groups with defects at the interface between the amnioserosa and lateral epidermis: a wavy dorsal opening, a round dorsal opening, and tearing along the amnioserosa/lateral epidermis border. The asterisk indicates that Df(2R)40 has a severe dorsal closure phenotype because of a lesion that falls outside of the mapped Df (see text). In this table, we refer to the Dfs by the screen name; the corresponding Bloomington stock number can be found in Appendix A.

### Amnioserosa Phenotypes (20 Dfs)

**Figure 3:**
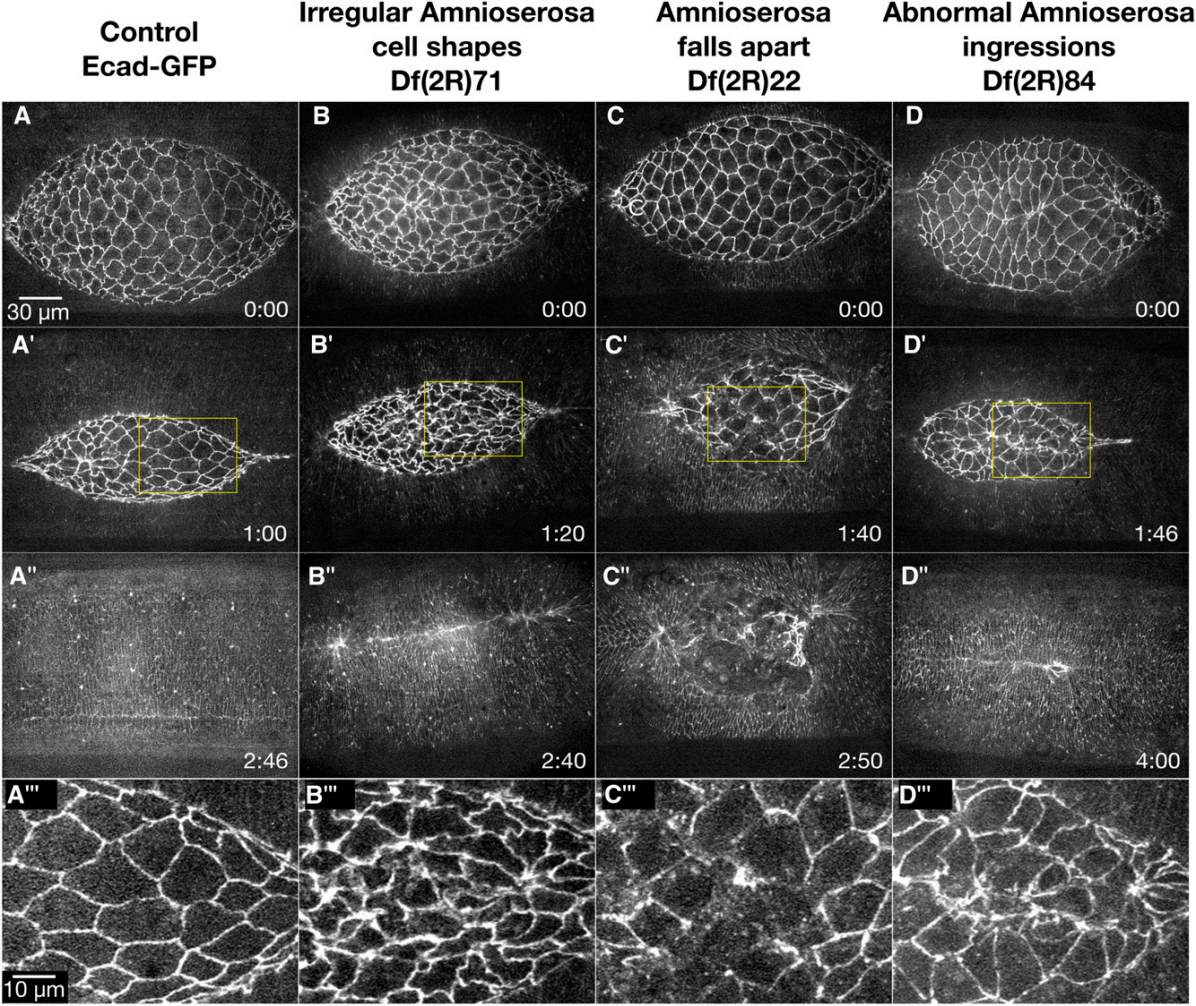
Amnioserosa Phenotypes. Time-lapse image series of Ecad-GFP labeled control embryos (A-A’’’) and homozygous Df embryos with amnioserosa phenotypes. Df(2R)71 embryos show irregular amnioserosa cell shapes (B-B’’’). Df(2R)22 embryos show an amnioserosa cell sheet that falls apart (C-C’’’). Df(2R)84 embryos show abnormal amnioserosa cell ingressions (D-D’’’). A’’’, B’’’, C’’’, and D’’’ show magnified views of the yellow boxed areas in the corresponding panels. Anterior is to the left, posterior to right. Time is in hr:min. The scale bar in A applies to panels A-D” (30 µm). The scale bar in A’’’ applies to panels A’’’-D’’’ (10 µm).

The squamous amnioserosa cells that fill the dorsal opening during closure have a characteristic shape, oscillate and ingress. By comparing these characteristics in control *vs.* homozygous Df embryos, we could identify genes whose products contribute to these features of closure. The amnioserosa cells in early closure are isotropic and their cell areas oscillate as the cells contract and relax (Fernández *et al.* 2007; Gorfinkiel *et al.* 2009; Blanchard *et al.* 2010; Sokolow *et al.* 2012). Early in closure, a large fraction of the amnioserosa cells have “wiggly” cell borders, suggesting that the borders are not under tension (Figure 1A’). The cell borders straighten out, but the overall cell shapes remain fairly isotropic throughout the bulk of closure (Figure 3A-A’’’) and as the tissue contracts, the amnioserosa cells have dampened oscillations. Throughout closure, cells are extruded (ingress) primarily from the amnioserosa sheet of cells at the canthi and from a region adjacent to the DME and PAS cells, termed the “marginal amnioserosa cells” (Fernández *et al.* 2007; Solon *et al.* 2009; Sokolow *et al.* 2012). A small fraction of the amnioserosa cells ingress from the bulk of the cell sheet. Even as amnioserosa cells ingress, cells in the surrounding cell sheet remain intact. The amnioserosa is affected in embryos homozygous for 20 of the 2R Dfs. The phenotypes are classified as irregular amnioserosa cell shapes, amnioserosa falls apart, and abnormal amnioserosa ingressions (Figure 3 and Table 1). Note that we believe we have also identified Dfs with amnioserosa oscillation defects, however, we did not consistently image at a frame rate that would allow us to unambiguously identify oscillation defects. We analyzed three of the Dfs (Df(2R)14, Df(2R)52 and Df(2R)66) suspected to cause abnormal oscillations at a frame rate of 30 sec, and found that two of the three Dfs (Df(2R)14 and Df(2R)52) cause significant oscillation defects. Thus, we cannot confidently classify amnioserosa oscillation defects at our standard two minute frame rate used in the screen. In Appendix A we report the two Dfs that cause oscillation defects as “confirmed” and the remainder of the Dfs that low time resolution time-lapse records suggest cause oscillation defects as “suspected”.

#### Irregular amnioserosa cell shapes:

The most common amnioserosa phenotype, seen in 16 2R Dfs, is varying degrees of irregular amnioserosa cell shapes (Table 1). Figure 3B shows an example of the defects in cell shape in Df(2R)71. In early closure, the cells are isotropic, similar to cells in control embryos. As closure progresses, the cells take on irregular shapes becoming anisotropic, stretching along the anterior-posterior axis (Figure 3B’’’). In addition, the cadherin fluorescence also appears thicker in many amnioserosa cells. The rate of closure in these embryos is comparable to control embryos, although the dorsal opening becomes slightly cigar-shaped suggesting that zipping is slowed. Four of 6 homozygous Df(2R)71 embryos imaged also have scarring at the dorsal midline post-closure, further suggesting abnormal zipping. It is unknown if the zipping defect observed in Df(2R)71 is due to the irregular amnioserosa shapes, the aberrant thickness of the cell junctions, some independent defect(s), or a combination of the three. These Df phenotypes, like all those described below, will be more appropriately investigated and interpreted once the gene(s) responsible within the Df are identified. Such studies will provide a better understanding of the molecular mechanisms that contribute to these aspects of dorsal closure.

#### Amnioserosa cells fall apart:

Throughout dorsal closure, the amnioserosa cells maintain adhesion with neighboring cells, even when individual cells ingress from the plane of the amnioserosa and apoptose (Kiehart *et al.* 2000; Fernández *et al.* 2007; Toyama *et al.* 2008; Gorfinkiel *et al.* 2009; Solon *et al.* 2009; Blanchard *et al.* 2010; Sokolow *et al.* 2012; Saravanan *et al.* 2013). In six 2R Dfs imaged, the amnioserosa cells or cell sheets fall apart, leading to large holes in the amnioserosa (Table 1). Embryos homozygous for Df(2R)22 provide an example of the amnioserosa cell sheet falling apart. The phenotype appears to primarily affect the amnioserosa, with the lateral epidermis and canthi remaining nearly indistinguishable from controls (Figure 3C-C’’’). At the start of closure, all seven embryos imaged appear normal (Figure 3C). In early to late closure, 4 of 7 embryos have an increased number of cells ingressing from the middle of the amnioserosa. Subsequently, junctional belts from the surrounding cells tear and the amnioserosa falls apart (Figure 3C’, C’’’). All seven embryos have an irregularly shaped dorsal opening; remarkably, 6 of 7 embryos complete closure, although four of these have scarring in the dorsal epidermis that forms post-closure (not shown). In the one embryo that did not complete closure, the amnioserosa cells fell apart in early closure and the leading edge recoiled away from the dorsal midline leaving a large hole filled with cellular debris (Figure 3C’’).

#### Increased amnioserosa cell ingressions:

In wild-type embryos >90% of ingressions occur at or near the purse string and the canthi, while less than 10% of cells ingress from the interior of the amnioserosa (Kiehart *et al.* 2000; Fernández *et al.* 2007; Toyama *et al.* 2008; Sokolow *et al.* 2012; Kiehart *et al.* 2017). Homozygous embryos of three different Dfs show abnormal amnioserosa ingressions from the interior or “bulk” of the dorsal opening (Table 1). Df(2R)84 has an average of 47 internal ingressions throughout closure compared to controls averaging four ingressions. This translates to an average rate of 0.35 ± 0.10 cells/min for internal amnioserosa cell ingression compared to controls averaging 0.05 ± 0.01 cells/min (*P* = 0.0302, N = 3 for both genotypes). The increased ingression disrupts amnioserosa cell shapes (Figure 3D’). In addition, the dorsal opening becomes misshapen in mid- to late-closure, although it is not clear if this is due to the increased ingression or is due to a different mechanism. Df(2R)84 also has a slowed closure rate of 11.1 ± 1.6 nm/sec compared to an average of 16.4 ± 1.8 nm/sec (for Ecad-GFP labeled embryos, *P* = 0.0172, N = 3 for both genotypes). Moderate increases in the rate of amnioserosa cell ingression and apoptosis speeds up closure (Toyama *et al.* 2008), but if rates of ingression are too high, closure fails. We surmise that the ingression rate observed in Df(2R)84 embryos is too high to sustain amnioserosa cell sheet integrity and normal rates of closure. It is conceivable that either the irregular cell shapes or the hypothesized decrease in the mechanical integrity of the amnioserosa cell sheet due to increased ingressions could cause closure to slow, but there remains the possibility that the two are unrelated phenotypes. Once the gene(s) responsible for cell shape and ingression phenotypes is/are identified, the molecular mechanism may be better understood.

### Lateral epidermis phenotypes (15 Dfs)

**Figure 4:**
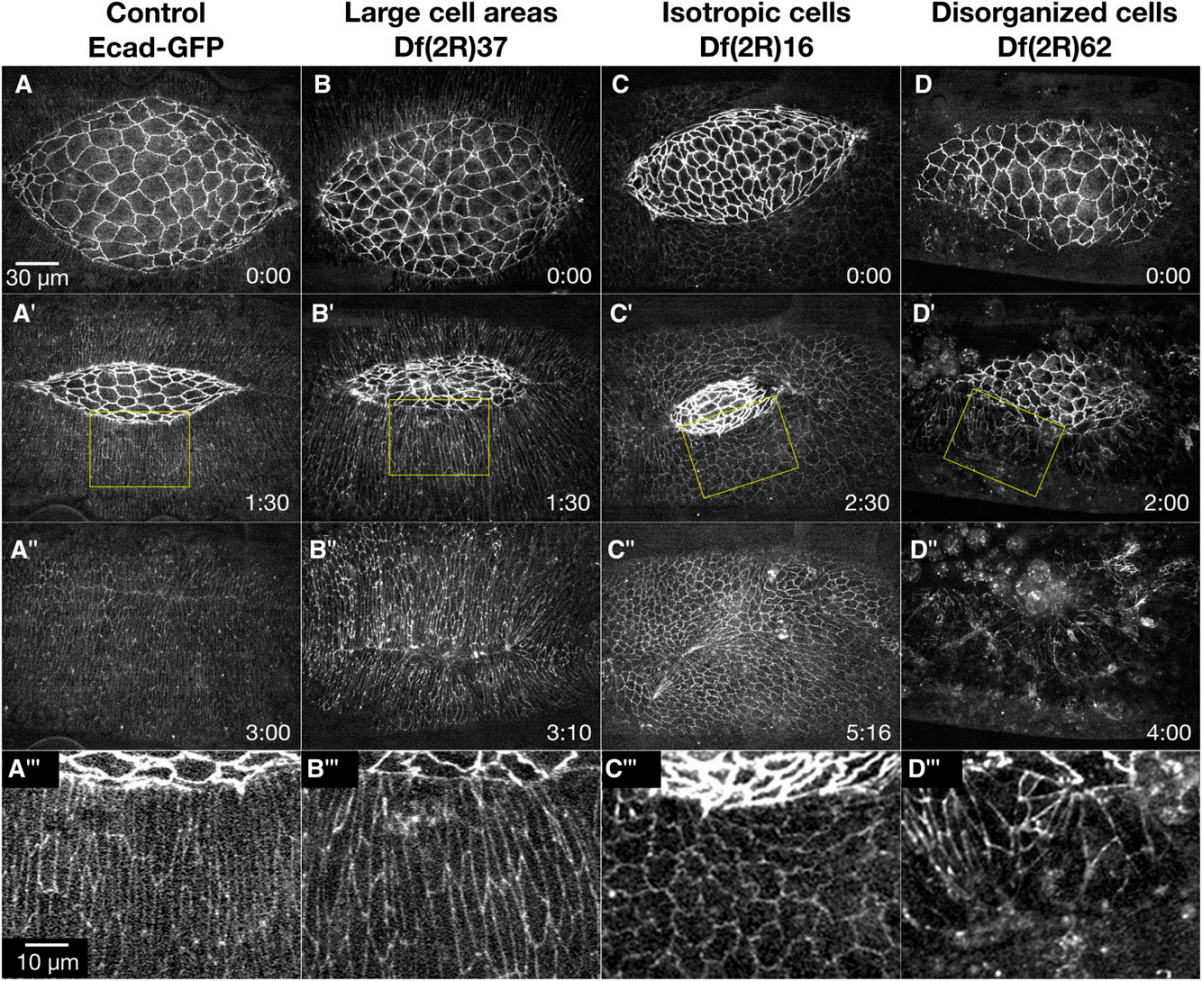
Lateral epidermis phenotypes. Time-lapse image series of embryos labeled with Ecad-GFP control embryos (A-A’’’) and homozygous Df embryos with lateral epidermis phenotypes. Df(2R)37 embryos show lateral epidermal cells with large cell areas (B-B’’’). Df(2R)16 embryos show isotropic lateral epidermal cells or lateral epidermal cells elongated along the anterior-posterior axis (C-C’’’). Df(2R)62 embryos show disorganized lateral epidermal cells (D-D’’’). A’’’, B’’’, C’’’, and D’’’ show magnified views of the yellow boxed areas of the lateral epidermis and adjacent amnioserosa in the corresponding panels. Anterior is to the left, posterior to right. Time is in hr:min. The scale bar in A applies to panels A-D” (30 µm). The scale bar in A’’’ applies to panels A’’’-D’’’ (10 µm).

During germ band retraction, prior to dorsal closure, the lateral epidermal cells are isotropic and become slightly elongated along a circumferential, dorsal-ventral axis (Young *et al.* 1993; Kiehart *et al.* 2000; Kiehart *et al.* 2017). As the purse string forms at the onset of closure, the lateral epidermal cells become more elongated, starting with the DME cells (Young *et al.* 1993; Jacinto *et al.* 2002). As closure continues, elongation progresses to more ventral rows of lateral epidermal cells. Throughout all of closure, the lateral epidermal cells remain organized in rows and are similarly sized (Figure 4A’ and A’’’). Once canthi form, the DME cells from opposing flanks of lateral epidermal sheets zip together to form a well-organized seamed, then seamless epithelium (Figure 4A’’). Fifteen Dfs are classified as causing defects in the lateral epidermis. We subdivided these lateral epidermis phenotypes into three categories: large cell areas, isotropic cell shapes and disorganized cells (Figure 4 and Table 2).

#### Large lateral epidermal cells:

After cellularization of the early embryo, most epithelial cells undergo two additional cycles of cell division with a small subset undergoing a third cycle – the amnioserosa does not undergo additional cell divisions (Hartenstein and Campos-Ortega 1985; Foe 1989; Knoblich *et al.* 1994). These subsequent cycles of cell division increase cell number and decrease lateral epidermal cell size. While defects in cell size can be the result of defects in cell division, which occurs prior to dorsal closure, these phenotypes may inform the mechanics of closure in a field of cells with irregular sizes. Large lateral epidermal cells are observed in six Dfs (Table 2).

Df(2R)37 is an excellent example of a Df that causes a large cell phenotype (Figure 4B-B’’’). In all nine embryos imaged, many of the lateral epidermal cells appear larger than control cells (see below for quantification). Even with their larger sizes, the lateral epidermal cells still appear to elongate circumferentially along the dorsal-ventral axis. Closure completes in a similar time to the control embryos, suggesting the cell size of lateral epidermal cells does not greatly perturb the mechanics of closure. Nevertheless, all Df(2R)37 embryos have slowed zipping at the posterior canthus in mid- to late-closure and cells become bunched and remain so in the newly formed seam post-closure (Figure 4B’’). It is unclear if the zipping defect seen here is due to the large lateral epidermal cells or is an additional phenotype caused by the deletion of another gene or genes in the Df.

#### Isotropic lateral epidermal cells:

In control embryos, the lateral epidermal cells begin to elongate dorsal-ventrally before the onset of dorsal closure and continue to elongate throughout closure (Figure 4A-A’). Embryos homozygous for each of four different 2R Dfs exhibit non-elongated, isotropic lateral epidermal cells, which persist throughout closure (Table 2). Df(2R)16 embryos provide an example of Dfs that cause persistent, isotropic lateral epidermal cells that fail to elongate and indeed, the DME cells tend to be elongated along the anterior-posterior axis of the embryo (Figure 4B’’’). All six Df(2R)16 embryos imaged have large, isotropic lateral epidermal cells at the onset of dorsal closure (Figure 4C). No obvious dorsal-ventral elongation of the lateral epidermal cells occurs in these embryos throughout the duration of closure (Figure 4C’-C’’’). This phenotype is similar to the loss of polarity phenotype reported in embryos lacking *wingless* function (Mcewen *et al.* 2000; Kaltschmidt and Brand 2002; Morel and Arias 2004). Additionally, the canthi are not well formed in Df(2R)16, remaining rounded throughout closure. The atypical canthi morphology may be a result of the aberrant DME morphology or a defect in zipping, but remarkably in spite of these defects these embryos close (Figure 4C’’).

#### Disorganized lateral epidermal cells:

The lateral epidermal cells remain similarly sized and organized in dorsal-ventrally elongated columns throughout dorsal closure in control embryos (Figure 4A-A’’’). Embryos homozygous for eight different 2R Dfs have disorganized lateral epidermal cells in which the cells elongate in both dorsal-ventral and anterior-posterior directions. An example of disorganized lateral epidermal cells is shown in an embryo homozygous for Df(2R)62 (Figure 4D-D’’’). All seven Df(2R)62 embryos imaged have disorganized lateral epidermal cells that fail to stretch toward the dorsal midline. The amnioserosa cells appear to behave normally, but zipping is slowed or inhibited. The two lateral epidermal sheets instead move toward a central point at the dorsal midline and the resulting dorsal epithelium is puckered (Figure 4D’’). The leading edge of the lateral epidermis remains scalloped throughout closure, and the anterior end of the amnioserosa is covered by hemocytes, which are macrophage-like cells that are frequently attracted to tissues with genetically or mechanically induced defects in the embryonic tissues (Rodriguez-Diaz *et al.* 2008; Wood and Martin 2017).

Df(2R)62 removes the gene *enabled* (*ena*) which was previously shown to be involved in dorsal closure. Cuticle preps of a loss-of-function *ena* allele (*ena^23^*) have mild dorsal puckering defects in 5–20% of embryos (Grevengoed *et al.* 2001; Gates *et al.* 2007). Removal of both the maternal and zygotic *ena* show increased head involution defects but defects in dorsal closure due to maternal and zygotic depletion of *ena* are similar to those seen in embryos with only zygotic loss of expression (Gates *et al.* 2007). Live imaging of homozygous *ena^23^* embryos display defects in segment alignment during zipping, which leads to scarring, but the lateral epidermal cells appear organized. Since both zygotic and maternal/zygotic knockdown of *ena* results in a much weaker phenotype than that seen in Df(2R)62, we conclude that the more severe Df phenotype is due to the effects of removing one or more genes, that in addition to *ena*, contribute to closure.

### Canthi/zipping phenotypes (32 Dfs)

**Figure 5:**
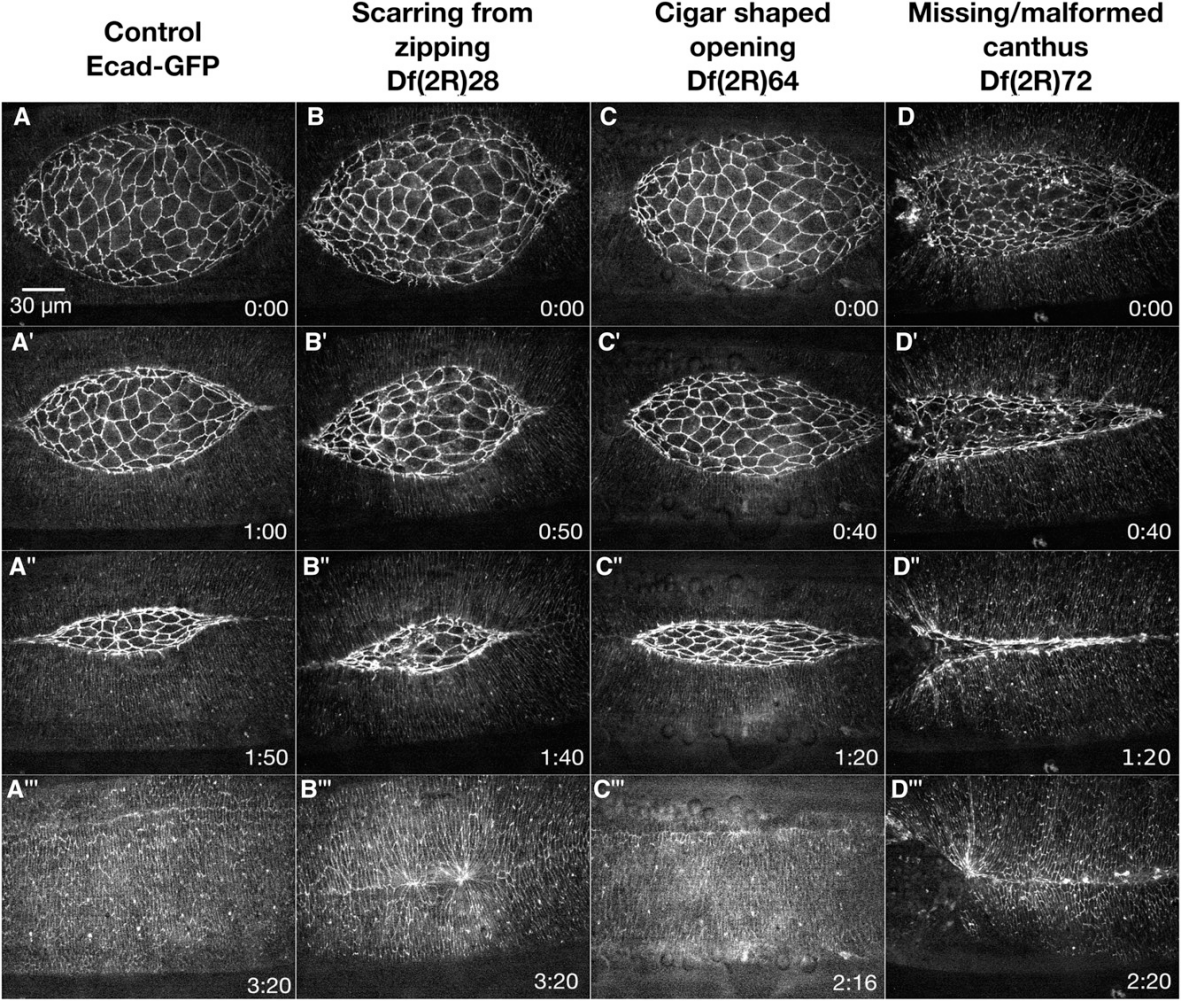
Zipping/Canthus phenotypes. Time-lapse image series of Ecad-GFP labeled control embryos (A-A’’’) and homozygous Df embryos with zipping/canthus phenotypes. Df(2R)28 embryos show scarring in the dorsal epidermis (B-B’’’). Df(2R)64 embryos show a cigar shaped opening (C-C’’’) and Df(2R)72 embryos show a missing canthus (D-D’’’). Anterior is to the left, posterior to right. Time is in hr:min. The scale bar in A applies to all micrographs (30 µm).

At the onset of closure, canthi form and the flanking sheets of lateral epidermal cells start to zip together at the anterior and posterior side poles of the previously ellipsoid-shaped dorsal opening. The ratio of height and width remains fairly constant throughout closure, an emergent property that maintains the dorsal opening in an eye shape with a fairly constant purse string curvature until the very end stages of closure (Figure 5A-A”; Hutson *et al.* 2003; Jankovics and Brunner 2006; Peralta *et al.* 2007). As closure progresses, the DME cells move into the canthus and zip together with the DME cells from the opposite sheet of lateral epidermis, eventually leaving a seamed, then seamless dorsal epithelium. The widths of the lateral epidermal cells measured along the seam are nearly uniform (Kiehart *et al.* 2000) and oscillate much like the amnioserosa cells (Peralta *et al.* 2008; Hunter *et al.* 2014). Actomyosin appears to play a role in these oscillations and in drawing the purse strings in a zipping step (Franke *et al.* 2005; Peralta *et al.* 2008; Lu *et al.* 2015). The purse string is thought to provide a taut leading edge for proper zipping (Jacinto *et al.* 2002). In addition, filopodia and lamellipodia are also involved in effective zipping (Jacinto *et al.* 2000; Gates *et al.* 2007; Millard and Martin 2008; Eltsov *et al.* 2015). Through the interplay of JNK, segmentation and anterior-posterior transcriptional cascades/signaling pathways, each DME cell at the leading edge of the lateral epidermal sheets is transcriptionally distinct from its neighbor. Perturbations in cell identity and differentiation due to these processes result in mis-alignment of matching segments (Perrimon and Desplan 1994; Mallo and Alonso 2013; Rousset *et al.* 2017). Thirty-two Dfs cause phenotypes affecting zipping and or the canthi. These are grouped as having scarring from zipping, a cigar-shaped dorsal opening, and/or missing or malformed canthi.

#### Scarring from zipping defects:

We define scarring as an irregular-shaped seam due to the fusion of lateral epidermal sheets with non-uniform widths of lateral epidermal cells where some cells are constricted and others are splayed out (Figure 5B’’’). Scarring is likely due to aberrant adhesion or zipping between DME cells at the leading edge of the advancing lateral epidermal cell sheets. In scarred embryos, the transition from a seamed to a seamless dorsal epithelium fails to occur properly. Embryos homozygous for many different Dfs have irregular-shaped lateral epidermal cells which results in scarring. Fourteen Dfs have normally-shaped lateral epidermal cells before zipping, but irregular-shaped lateral epidermal cells form during or after zipping. In these Dfs, the scarring is likely due to defects in zipping and/or adhesion, and not the shape of the lateral epidermal cells before zipping.

An example of scarring is seen in embryos homozygous for Df(2R)28. Df(2R)28 embryos start closure with normally shaped amnioserosa and lateral epidermal cells (Figure 5B). In mid- to late-closure, 9 of 12 embryos have some irregular amnioserosa cell shapes, five of these may be caused by increased cell ingression from the bulk of the amnioserosa (Figure 5B’). All twelve embryos complete closure, although all embryos with a visible seam at the end of closure have scarring (Figure 5B’’’). Df(2R)28 removes the serine/threonine kinase-encoding homolog of the mammalian oncogene *Mos oncogene* (*Mos*). *Mos* was reported as having defects in segmentation, dorsal closure and head involution when knocked down via transgenic RNAi (Sopko *et al.* 2014). However, *Mos* is also removed by Df(2R)29 which has no detectable phenotype, including a normal seam (data not shown). This suggests the deletion of *Mos* does not cause the observed phenotype in Df(2R)28 but it is instead caused by the deletion of one or more other genes. Alternatively, the breakpoints that define Df(2R)29 may not have been properly identified. In addition, the *Mos* defect was reported via cuticle preparations, which are not directly comparable to our Df images. Moreover, our homozygous Df embryos are zygotically null for *Mos*, so may be more (or possibly less) severe than that caused by RNAi mediated knock down.

#### Cigar-shaped dorsal opening:

With the formation of the canthi in early closure, the dorsal opening forms a characteristic eye shape. An emergent consequence of zipping is that changes in the width of the dorsal opening are closely coordinated with changes in the height of the dorsal opening. This emergent, coordination maintains a nearly constant curvature of the purse strings and the eye shape of the dorsal opening throughout closure (Figure 5A-A’’). Inhibition of zipping results in a cigar-shaped dorsal opening (Hutson *et al.* 2003; Jankovics and Brunner 2006). Defective, cigar-shaped dorsal openings were identified in embryos homozygous for eleven different 2R Dfs (Table 3).

Embryos homozygous for Df(2R)64 provide an excellent example of defects in closure that cause a cigar-shaped dorsal opening (Figure 5C-C’’’). Canthi form in all seven Df(2R)64 embryos imaged, resulting in a normal eye-shaped dorsal opening at the beginning of closure (Figure 5C). By mid- to late-closure, the opening becomes cigar-shaped because zipping appears to stall (Figure 5C’-C’’). All Df(2R)64 embryos complete closure without scarring in the DME (Figure 5C’’’). This indicates that despite slowed zipping, cell sheet alignment was not perturbed. Interestingly, all Df(2R)64 embryos imaged in early closure have less dynamic amnioserosa oscillations (see Table 1), but because multiple genes are removed in this Df, it is unclear if the oscillation and zipping phenotypes are related.

#### Missing/malformed canthus:

Although the Dfs with cigar-shaped dorsal openings have slowed zipping, the overall shape of their canthi appear normal, indicating correct formation of the canthi. An overlapping group of phenotypes, missing or malformed canthi, was identified in embryos homozygous for thirteen different 2R Dfs. Df(2R)72 is an example of a Df which rarely forms an anterior canthus (Figure 5D-D’’’). All six of the embryos imaged have a zipping/canthus defect at the anterior end. A single Df(2R)72 embryo forms an anterior canthus, but zipping is still slowed. The other five embryos do not form an anterior canthus and all zipping is from the posterior end (Figure 5D’-D’’). Some of these embryos have a large hole anterior to the dorsal opening, indicating additional defects in head involution (Figure 5D’’’).

Df(2R)72 removes *shotgun* (*shg*) which encodes the cell adhesion protein E-cadherin. Deletion of *shg* has previously been shown to cause defects in dorsal closure and head involution. A null allele, *shg^R64^*, has reduced levels of E-cadherin in the lateral epidermis but levels in the amnioserosa are comparable to control. These *shg^R64^* embryos have small dorsal holes and segment mismatches as a result of misalignment during dorsal closure (Gorfinkiel and Martinez-Arias 2007). Recall that we image Df homozygous embryos using a ubiquitously expressed transgene that encodes Ecad-GFP to label cell junctions. This same Ecad-GFP construct was shown to completely rescue the lethality of *shg^R64^* (Oda and Tsukita 2001). We have confirmed that overexpression of Ecad-GFP completely rescues the dorsal closure phenotype observed in *shg* null embryos (see below). Because Df(2R)72 dorsal closure phenotypes are not completely rescued by Ecad-GFP we infer that Df(2R)72 removes an additional gene or genes that are required for the formation of the anterior canthus and successful closure. These additional genes may or may not work in concert with *shg*.

### Phenotypes at the interface between the amnioserosa and lateral epidermis (20 Dfs)

At the onset of closure, the leading edge of the lateral epidermis, formed by the DME cells, is initially scalloped. JNK signaling in the DME (Homsy *et al.* 2006) and expression of the transmembrane protein Echnioid in the DME, but not the amnioserosa (Laplante and Nilson 2006; Laplante and Nilson 2011) is necessary for the accumulation of actomyosin-rich purse strings in the DME cells that resolves the scalloped leading edge into a smooth arc (Figures 1A”- 1B”, 6A’ and Kiehart *et al.* 2000). In addition to the signaling that occurs between the DME and PAS cells for the proper establishment of the purse strings, the DME and PAS cells become reciprocially wedge-shaped as the PAS cells move underneath the DME cells. Integrins are essential for the remodeling of these cells. Throughout closure, the DME cells maintain this interaction with the PAS cells until they move into the canthus, where the DME cells remodel during zipping to adhere to the opposing DME cells and the amnioserosa cells internalize and apoptose (Wada *et al.* 2007; Rodriguez-Diaz *et al.* 2008; Lu *et al.* 2015; Kiehart *et al.* 2017). Both integrin-mediated adhesions and adherens junctions are essential for the integrity of the connection between the DME and PAS cells (Kaltschmidt *et al.* 2002; Hutson *et al.* 2003; Gorfinkiel and Martinez-Arias 2007; Mateus and Martinez Arias 2011).

**Figure 6:**
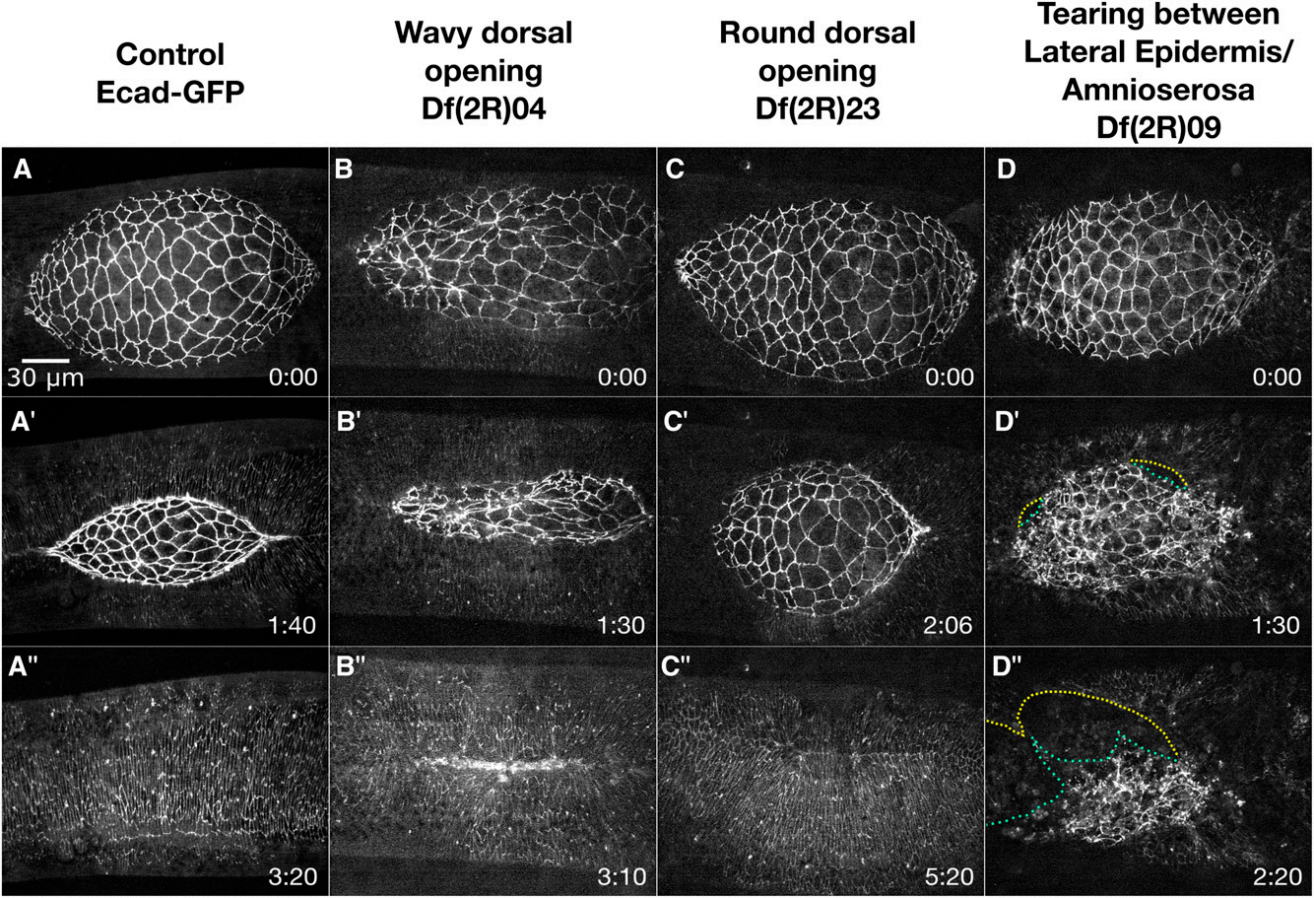
Phenotypes at the interface of the amnioserosa and lateral epidermis. Time-lapse image series Ecad-GFP labeled control embryos (A-A’’’) and homozygous Df embryos with defects at the interface of the lateral epidermis and amnioserosa. Df(2R)04 embryos show a dorsal opening with a wavy edge (B-B’’’). Df(2R)23 embryos show a round dorsal opening (C-C’’’). Df(2R)09 embryos show tearing between the amnioserosa and lateral epidermis (D-D’’’). The yellow line shows the lateral epidermis edge and the green line shows the amnioserosa edge (D’ and D’’). Anterior is to the left, posterior to right. Time is in hr:min. The scale bar in A applies to all micrographs (30 µm).

A number of homozygous Dfs cause dorsal closure phenotypes which affect the interface between the amnioserosa and lateral epidermis and result in aberrantly shaped dorsal openings (wavy or rounded) or tearing along the border between the DME and PAS cells.

#### Wavy dorsal opening:

A persistently scalloped or wavy dorsal opening phenotype is likely to result from disrupted or weak purse strings or from increased forces from the lateral epidermis or the amnioserosa. Such phenotypes characterize embryos homozygous for nine 2R Dfs (Table 4). Embryos homozygous for Df(2R)04 provide an example of a Df with a persistent wavy dorsal opening, observed in all seven embryos imaged. The edge of the lateral epidermis remains slightly scalloped throughout closure unlike the smooth arc seen in control embryos in mid-closure (Figure 6B’ compared to A’). Additionally, the shapes of the amnioserosa cells are irregular in all Df(2R)04 embryos imaged. Embryos also have a cigar-shaped dorsal opening indicating zipping is slowed. All embryos complete closure with scarring in the formed, dorsal epidermis (Figure 6B’’). To evaluate the nature of the purse string in this Df, the next step will be to use an imaging background that encodes fluorescent tags for F-actin and myosin.

Df(2R)04 removes the Src family tyrosine kinase encoding gene *Src oncogene at 42A* (*Src42A*). Strong mutants of *Src42A* cause scarring in the lateral epidermis at the end of closure and show a more severe dorsal open phenotype when disrupted together with other Src family genes (Tateno *et al.* 2000; Murray *et al.* 2006). *Src42A* is also removed by Df(2R)03 which has irregularly shaped amnioserosa cells and scarring. Df(2R)03 has a smoothly curved dorsal opening such that the wavy phenotype is considerably less severe than that seen in Df(2R)04. Therefore, we conclude that part of the phenotype in Df(2R)04, specifically the wavy dorsal opening, is due to deletion of another gene besides the *Src42A* gene.

#### Round dorsal opening:

We define a round or rounded dorsal opening as an opening with a width (measured from canthus to canthus along the anterior-posterior axis) similar to the height (measured along the circumferential dorsal-ventral axis). This phenotype was identified in embryos of four different 2R Dfs (Table 4). These phenotypes may be due to faster zipping, decreased amnioserosa contraction, or a combination of the two.

Embryos homozygous for Df(2R)23 provide an example of a Df with a round dorsal opening, observed in all seven embryos imaged (Figure 6C-C’’). Embryos start closure looking normal with robust amnioserosa oscillations. Shortly after the onset of closure, the oscillations and dorsal opening become relaxed and the whole dorsal opening becomes rounder than in control embryos (Figure 6C’ compare with Figure 6A’). The cell areas of the central amnioserosa cells remain large until late in closure. Zipping slows later in closure, but still completes with noticeable scarring in only 2 of 7 embryos. Cadherin belts between PAS cells also extend (abnormally) under the lateral epidermis (see below), indicating a defect in the DME/PAS interface. Additionally, the yolk that is normally pushed lower into the embryo remains near the epithelial cells throughout the duration of and after closure (data not shown).

#### Tearing Between Lateral Epidermis/Amnioserosa:

During normal closure, the DME cells of the lateral epidermis and the PAS cells of the amnioserosa remain in contact with each other until the end of closure (Jacinto *et al.* 2000; Hutson *et al.* 2003; Wada *et al.* 2007; Lu *et al.* 2015; Kiehart *et al.* 2017). As described above, cadherin-based adherens junctions and integrins are necessary to maintain interaction between these two tissues (Kaltschmidt and Brand 2002; Hutson *et al.* 2003; Narasimha and Brown 2004; Homsy *et al.* 2006; Gorfinkiel and Martinez-Arias 2007; Peralta *et al.* 2007; Mateus and Martinez Arias 2011; Jurado *et al.* 2016). The 2R Df screen identified nine Dfs that when homozygosed, cause tearing between the DME and PAS cells. Remarkably, this tearing does not always result in the failure of closure, particularly if it occurs in mid- to late-closure.

Embryos homozygous for Df(2R)09 provide an example of this tearing phenotype. In all eight embryos imaged, the shape of the dorsal opening and of individual amnioserosa cells are comparable to control embryos at the onset of closure (Figure 6D). In contrast, the lateral epidermis at this stage shows signs of disorganization in 5 of 8 embryos (not shown). By mid-closure, small tears form between the DME and PAS cells in each of these five embryos (Figure 6D’). In addition, the amnioserosa cells also become irregularly shaped. The tears between the DME and PAS cells expand as the lateral epidermis pulls away from the dorsal midline (Figure 6C’’). The tears become large enough to allow yolk from the interior of the embryo to pour into the perivitelline space, and subsequently closure fails. Of the three embryos imaged for longer duration, all three fail in closure, leading us to conclude that this Df causes tearing between the DME and PAS and results in a failure of closure.

Three embryos homozygous for Df(2R)09 do not show signs of tearing or disorganized lateral epidermal cells and all complete closure. Two of these embryos have irregularly shaped amnioserosa cells and result in scarring of the epithelium at the dorsal midline after closure completes. The third embryo is indistinguishable from control embryos. We hypothesize that the gene product(s) responsible for this phenotype are likely maternally loaded and that unequal perdurance of these maternally loaded products leads to the decreased penetrance of the phenotype.

### Dfs not imaged

We were unable to image four 2R Dfs in dorsal closure. Df(2R)30 lays unfertilized eggs when balanced with TGC. This Df overlaps nine other Dfs in the 2R Df kit, including 3 which fail in dorsal closure. Any additional phenotypes due to the four genes removed by this Df and not by the other nine overlapping Dfs would be masked by the severity of the phenotypes seen in the three overlapping Dfs that fail in closure. Three Dfs (Df(2R)77, Df(2R)80, and Df(2R)89) are maintained as stocks by balancer chromosomes lacking *Cy*, making them difficult to distinguish in our crossing scheme. They also all remove ribosomal proteins which give dominant *Minute* phenotypes and lead to several defects including poor fertility and viability (Marygold *et al.* 2007). In these stocks, the balancers also contain duplications to rescue the *Minute* phenotype. Altogether, these four Dfs remove 34 genes not removed by other Dfs in the kit. Due to the complex nature of these Dfs and the small number of untested genes they remove, we are currently not pursuing them further. A truly saturating screen for dorsal closure genes will need to investigate these 34 genes.

### Dfs with published dorsal closure genes

Thirty-seven Dfs remove genes that were previously identified as “dorsal closure genes”, *i.e.*, genes known to affect dorsal closure when deleted or knocked down via RNAi. Six of these Dfs cause closure phenotypes similar to the published descriptions of the dorsal closure genes they remove. Twelve Dfs cause stronger phenotypes than the published phenotypes of the dorsal closure genes they remove, suggesting these Dfs remove additional genes that affect closure. Two Dfs remove the same gene, *Zasp52*, and cause a phenotype that is less severe than the reported gene phenotype (Ducuing and Vincent 2016). Further investigation of the *Zasp52*Δ chromosome used in that study suggests that the more severe dorsal closure phenotype is due to a 2^nd^ site lesion that is not associated with the *Zasp52* locus (R. D. Mortensen, S. M. Fogerson, H. Y. Chiou, J. M. Crawford, D. P. Kiehart unpublished data). Nine Dfs cannot be directly compared to the phenotypes reported by the genes removed, for multiple reasons (*e.g.*, the previous studies did not include live imaging analysis, the mutations studied were not null, or were investigated using RNAi knock-down animals, which may also fail to phenocopy the zygotic null phenotypes characteristic of homozygous Dfs, see Table 5 and Appendix A for more details). Eight Dfs which remove dorsal closure genes cause no identifiable phenotype. This is not surprising because the genes removed by these Dfs show a phenotype only in germ-line clones, in combination with other mutants, or have very low penetrance (Table 5 and Appendix A).

**Table 5:** Deficiencies with published dorsal closure genes

Screen Name	Known Dorsal Closure Gene(s)	References	Known gene fully phenocopies Df?	Comments
Df(2R)02	*scaf*	(Rousset *et al.* 2010) (Sorrosal *et al.* 2010)	Yes	Similar to published phenotype
Df(2R)03	*Src42A*	(Tateno *et al.* 2000)	Unknown	Phenotype described from cuticle defects in combination with other mutants
Df(2R)04	*Src42A*	(Tateno *et al.* 2000)	No	Phenotype described from cuticle defects in combination with other mutants, Df(2R)04 is more severe than Df(2R)03
Df(2R)10	*ptc*	(Jankovics *et al.* 2011)	No	Published phenotype is less severe
Df(2R)11	*Gγ1*, *ptc*	(Yi *et al.* 2008) (Jankovics *et al.* 2011)	Unknown	Additive effect of 2 genes has not been tested
Df(2R)12	*Pkn*	(Lu and Settleman 1999)	Unknown	Phenotype described from germline clones and cuticle defects
Df(2R)17	*Jra*	(Riesgo-Escovar and Hafen 1997)	No	Published phenotype is less severe
Df(2R)18	*Jra*	(Riesgo-Escovar and Hafen 1997)	Yes	Similar to published phenotype
Df(2R)20	*shn*, *acal*	(Rusten *et al.* 2002) (Fernandez *et al.* 2007) (Ríos-Barrera *et al.* 2015)	Unknown	Additive effect of 2 genes has not been tested
Df(2R)21	*shn*	(Rusten *et al.* 2002) (Fernandez *et al.* 2007)	No	Published phenotype is less severe
Df(2R)28	*Mos*	(Sopko *et al.* 2014)	No	Phenotype described from cuticle defects with RNAi, Df(2R)28 is more severe than Df(2R)29
Df(2R)29	*Mos*	(Sopko *et al.* 2014)	Unknown	Phenotype described from cuticle defects with RNAi
Df(2R)32	*Ack-like*	(Zahedi *et al.* 2008)	No	Published phenotype is less severe
Df(2R)33	*GstE14*	(Enya *et al.* 2014)	Unknown	Phenotype described from cuticle defects
Df(2R)35	*shot*	(Takács *et al.* 2017)	No	Published phenotype is less severe
Df(2R)36	*shot*	(Takács *et al.* 2017)	N/A	Problem with Df (see below)
Df(2R)37	*shot*	(Takács *et al.* 2017)	No	Published phenotype is less severe
Df(2R)44	*scb*, *Arf51F*	(Wada *et al.* 2007) (Jankovics *et al.* 2011)	Unknown	Additive effect of 2 genes has not been tested
Df(2R)45	*Zasp52*	(Ducuing and Vincent 2016)	No	*Zasp52* published phenotype is more severe due to a suspected 2^nd^ site lesion
Df(2R)46	*Zasp52*	(Ducuing and Vincent 2016)	No	*Zasp52* published phenotype is more severe due to a suspected 2^nd^ site lesion
Df(2R)47	*Rho1*	(Lu and Settleman 1999) (Rodriguez-Diaz *et al.* 2008)	Yes	Similar to published phenotype
Df(2R)48	*shark*	(Fernandez *et al.* 2000)	No	Phenotype described from cuticle defects in germline clones, zygotic nulls survive to larval stages, Df does not hatch
Df(2R)50	*Cdk4*	(Sopko *et al.* 2014)	Unknown	Phenotype described from RNAi
Df(2R)52	*RhoGEF2*	(Azevedo *et al.* 2011)	Unknown	Phenotype described from germline clones
Df(2R)53	*RhoGEF2*	(Azevedo *et al.* 2011)	Unknown	Phenotype described from germline clones
Df(2R)57	*POSH*	(Lennox and Stronach 2010) (Zhang *et al.* 2010)	Unknown	Published phenotype described from cuticle defects, has low penetrance (10–12%)
Df(2R)62	*ena*	(Grevengoed *et al.* 2001) (Gates *et al.* 2007)	No	Published phenotype is less severe
Df(2R)63	*rib*, *cora*, *ena*	(Nüsslein-Volhard *et al.* 1984) (Blake *et al.* 1998) (Lamb *et al.* 1998) (Grevengoed *et al.* 2001) (Gates *et al.* 2007)	Unknown	Additive effect of 3 genes has not been tested
Df(2R)69	*mir-311*, *mir-312*	(Leaman *et al.* 2005)	Unknown	Phenotype described from RNAi
Df(2R)70	*mir-311*, *mir-312*	(Leaman *et al.* 2005)	Unknown	Phenotype described from RNAi
Df(2R)71	*mir-311*, *mir-312*	(Leaman *et al.* 2005)	No	Df(2R)71 has more severe phenotype than Df(2R)69 and 70
Df(2R)72	*shg*	(Gorfinkiel and Martinez-Arias 2007)	No	Ecad-GFP rescues null completely (see Fig 12 and 13)
Df(2R)75	*Egfr*	(Shen *et al.* 2013)	Yes	Similar to published phenotype
Df(2R)85	*gbb*	(Chen *et al.* 1998)	Unknown	Phenotype described in combination with other mutants
Df(2R)90	*zip*	(Jacinto *et al.* 2002) (Franke *et al.* 2005)	Yes	Similar to published phenotype
Df(2R)91	*Kr*	(Jankovics *et al.* 2011)	No	Published phenotype is less severe
Df(2R)92	*Kr*	(Jankovics *et al.* 2011)	Yes	Similar to published phenotype

Deficiency phenotypes were compared with the phenotype(s) of the previously identified dorsal closure gene(s) deleted by the Df. Column 1 refers to the assigned Df number for the screen; the corresponding Bloomington stock number can be found in Appendix A. Column 2 lists the previously identified dorsal closure gene(s) removed by the Df. The third column lists the references in which these dorsal closure genes were identified. The fourth column lists whether the phenotype of the dorsal closure gene fully phenocopies the homozygous Df. The fifth column summarizes how the phenotypes compare to published phenotypes.

### Identifying the individual genes that cause the Df’s dorsal closure phenotype

The phenotypes described above are for embryos homozygous for the Dfs and are due to the deletion of more than one gene. Establishing the molecular mechanisms responsible for the phenotypes observed requires identifying which deleted gene or genes are responsible for the observed phenotype(s). This can be done through imaging overlapping Dfs, duplications, and individual mutations. Some Dfs may delete an obvious candidate gene or genes that is (are) most likely to contribute to the Df phenotype. Using these strategies, we have already identified four novel genes affecting dorsal closure that contribute to the phenotypes caused by six deficiency kit Dfs. The dorsal closure phenotypes caused by two of these Dfs, Df(2R)16 and Df(2R)60, are each due to the deletion of a single gene. The phenotypes caused by homozygosing the other four Dfs, Df(2R)17, Df(2R)23, Df(2R)37 and Df(2R)61, are only partially caused by the mutation of these newly identified genes. We conclude that the deletion of an additional gene or genes in each Df contribute to its dorsal closure phenotype(s).

### The Df(2R)37 dorsal closure phenotypes are partially caused by shot and tum

**Figure 7:**
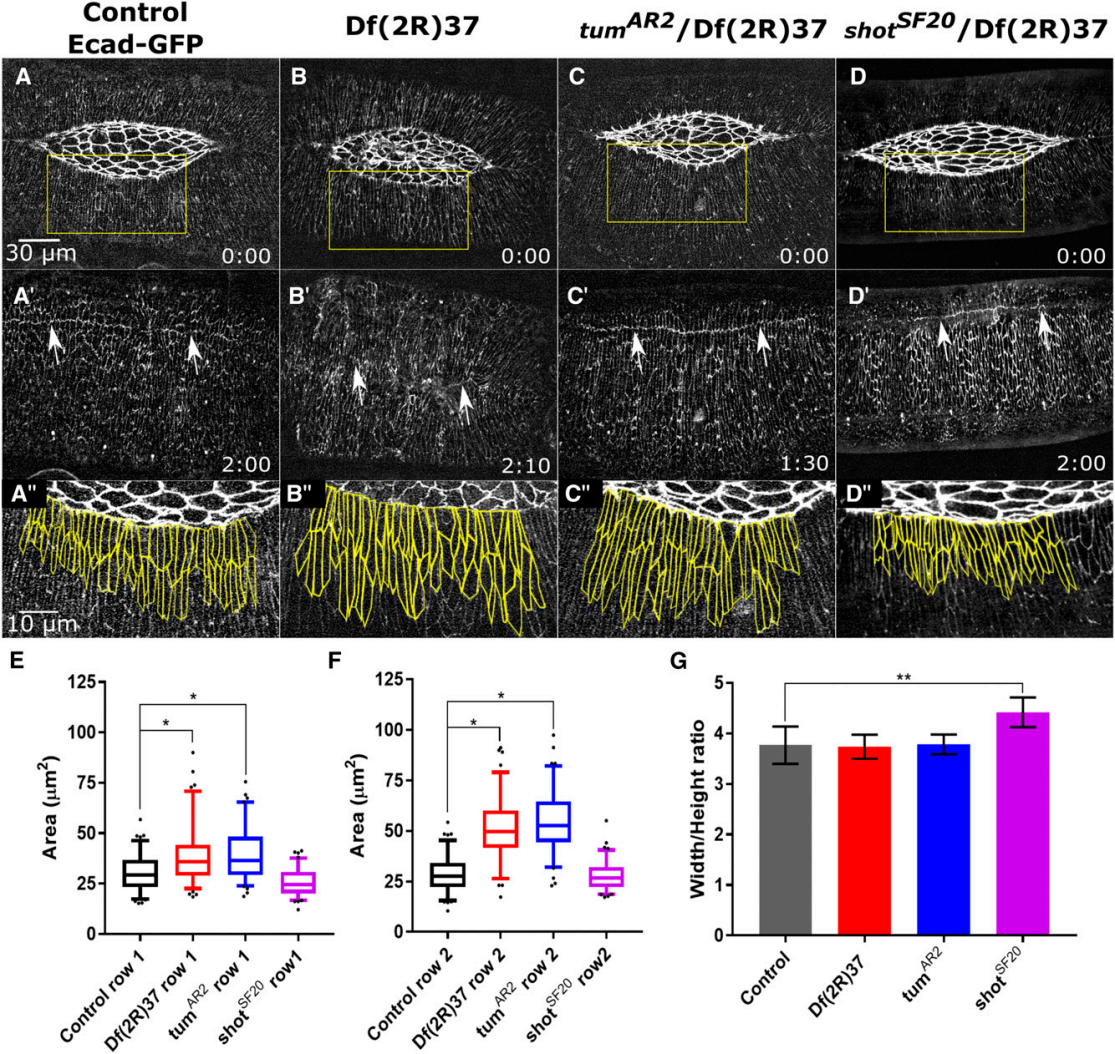
Deletion of *shot* and *tum* both contribute to different Df(2R)37 phenotypes. Time-lapse image series of a Ecad-GFP labeled control (A-A’’’), homozygous Df(2R)71 (B-B’’), *tum^AR2^*/Df(2R)37 (C-C’’’) and *shot^SF20^*/Df(2R)37 embryos (D-D’’’). The top row shows embryos in mid-dorsal closure, and the second row shows the seam of the same embryos after closure completes, indicated by arrows. A’’, B’’, C’’, and D’’ show magnified views of the yellow boxed areas of the lateral epidermis in the corresponding panels, and the yellow traces of the cell outlines of 20 cells in the 1^st^ and 2^nd^ rows of lateral epidermal cells for area quantification. Box plots show the median, upper and lower quartiles of the cell area distribution. Whiskers show 5% and 95% confidence intervals of the cell area distribution. E is for the 1^st^ row and F is for the 2^nd^ row of the lateral epidermal cell sheet. The 1^st^ and 2^nd^ rows of the lateral epidermal cells in Df(2R)37 and *tum^AR2^*/Df(2R)37 embryos have a significant increase in cell area in comparison to the control and *shot^SF20^*/Df(2R)37, thus the increase in cell size of Df(2R)37 is due to loss of tum. The Width/Height ratio with standard deviation shows slower zipping in *shot^SF20^*/Df(2R)37 than in control embryos (*P < 0.001 and **P < 0.05, G), while *tum^AR2^*/Df(2R)37 shows no significant difference to zipping in control embryos. Thus, loss of shot in Df(2R)37 causes the observed zipping defect in the Df. Anterior is to the left, posterior to right. Time is in hr:min. The scale bar in A applies to panels A-D’ (30 µm). The scale bar in A’’ applies to panels A’’-D’’ (10 µm).

An allele of the gene *short stop* (*shot*) was identified as having a zipping phenotype during dorsal closure at the time we were completing our screen of Dfs on 2R (Takács *et al.* 2017). The Shot protein is a member of the spectraplakin family of proteins and acts at cell junctions to link F-actin and microtubules. It contributes to effective zipping in dorsal closure as loss of *shot* leads to a cigar-shaped dorsal opening likely due to disrupted filopodia formation at the leading edge, which is important for appropriate cell matching at the canthi. We imaged the same *shot* allele, *shot^SF20^* crossed to Df(2R)37 in an Ecad-GFP background to see if it compares to the phenotype seen in Df(2R)37 (Figure 7D). Transheterozygous *shot^SF20^*/Df(2R)37 embryos have a slight cigar-shape to the dorsal opening, but this is much less severe than the published phenotype for *shot^SF20^* homozygous embryos.

To compare directly and quantitatively the phenotype of Df(2R)37 embryos to the published phenotype for *shot*, we compared the Width (anterior-posterior length)/Height (dorsal-ventral length) ratio of the embryos at 30µm Height as per Takács *et al.* 2017. Control embryos have a ratio of 3.80 ± 0.37 and *Shot^SF20^*/Df(2R)37 embryos have a ratio of 4.40 ± 0.29 (N = 6 embryos for both; Figure 7). While these are statistically significant, they are not as different as that published (Takács *et al.* 2017). This discrepancy may be due to the difference in embryos labeled with Ecad-GFP (which we used) *vs.* the zasp-EGFP (used in Takács *et al.* 2017). The phenotype seen in *shot* mutants does not phenocopy the large lateral epidermal cells and scarring observed in Df(2R)37 embryos.

We identified *tumbleweed* (*tum*) as a candidate removed by Df(2R)37 that might be responsible for the large cell phenotype. The *tum* gene encodes a racGAP protein shown by RNAi to be involved in cytokinesis in cell culture (Somers and Saint 2003; Echard *et al.* 2004). Embryos with a loss of function *tum* allele were subsequently shown to have defects in cell division prior to stage 11, leading to binucleate cells (Jones and Bejsovec 2005). Furthermore, *tum* is a negative regulator of the *wingless/Wnt* pathway, a major pathway in dorsal closure (Mcewen *et al.* 2000; Kaltschmidt *et al.* 2002; Morel and Arias 2004; Jones and Bejsovec 2005). We found embryos null for *tum* have larger lateral epidermal cells than control embryos. This is similar to the cell size defect observed in homozygous Df(2R)37 embryos (Figure 7C’’ compare to Figure 7B’’).

To further analyze the size of lateral epidermal cells in Df(2R)37 and *tum*/Df(2R)37 embryos, we quantified the cell areas of the first two rows of lateral epidermal cells measured at the level of the E-Cadherin-rich junctional belts. Specifically, we assessed area in the DME cells (row 1, Figure 7E) and the cells one row ventral of the DME cells (row 2, Figure 7F). Cell area in the first row of cells in homozygous Df(2R)37 embryos and in *tum*/Df(2R)37 embryos are slightly larger than in controls (39 ± 14µm^2^ and 39 ± 13µm^2^ respectively *vs.* controls, 31 ± 10 µm^2^, *P* < 0.0001, N = 6 Figure 7E). Interestingly, the cell areas in row 2 of homozygous Df(2R)37 and *tum*/Df(2R)37 embryos (51 ± 15µm^2^ and 54 ± 15µm^2^ respectively), are much larger than the cell areas of row 2 in control embryos (29 ± 9µm^2^, Figure 7F). Defects in cell division are expected to cause changes in cell volume and here, we have measured cell area. We surmise that the modest increase in row 1 cell area is due to morphological constraints on cell area and suspect that volumes of row 1 cells in homozygous Df(2R)37 or *tum*/Df(2R)37 embryos are considerably larger than their control counterparts. We also compared the Width/Height ratio of *tum*/Df(2R)37 embryos at 30µm Height and found they are similar to control embryos, 3.80 ± 0.02 and 3.80 ± 0.37 respectively. Surprisingly this ratio in homozygous Df(2R)37 is also similar to controls at 3.70 ± 0.24. This suggests that the zipping phenotype due to the deletion of *shot* is masked in the Df either due to the larger cell size, from deletion of *tum*, or the deletion of another gene in Df(2R)37.

Thus far, we have determined the deletion of the two genes *shot* and *tum* contribute to the phenotype seen in Df(2R)37. Although both of these gene mutants show dorsal closure phenotypes, neither results in the scarring observed in homozygous Df(2R)37 embryos (Figure 3B’’ and Figure 6B’). It is possible the scarring is an additive effect of the combined deletion of both *shot* and *tum* or that there is a third gene responsible for the more severe phenotype. The combined deletion of both genes through recombination to see if the phenotype seen in Df(2R)37 can be recapitulated would be difficult, as the genes are much, much less than one map unit apart. Another method to test if the phenotype seen in Df(2R)37 is an additive effect is to rescue the function of one or both of *tum* and *shot* genes. The *UAS -RacGap50C* insertion driven by E22C-Gal4 can rescue most *tum* mutant embryos through embryogenesis, but most die as larvae, likely because E22C-Gal4 is an embryonic driver (Jones and Bejsovec 2005). We have tested for rescue of the *tum* mutant phenotype in homozygous Df(2R)37 embryos that also express a UAS-RacGap50C transgene driven by daughterless-Gal4. Rescued embryos have small (wild type) cells, but still show scarring – we surmise that another gene or genes deleted by Df(2R)37 is responsible for the scarring phenotype (data not shown).

### The deletion of even-skipped phenocopies Df(2R)16

**Figure 8:**
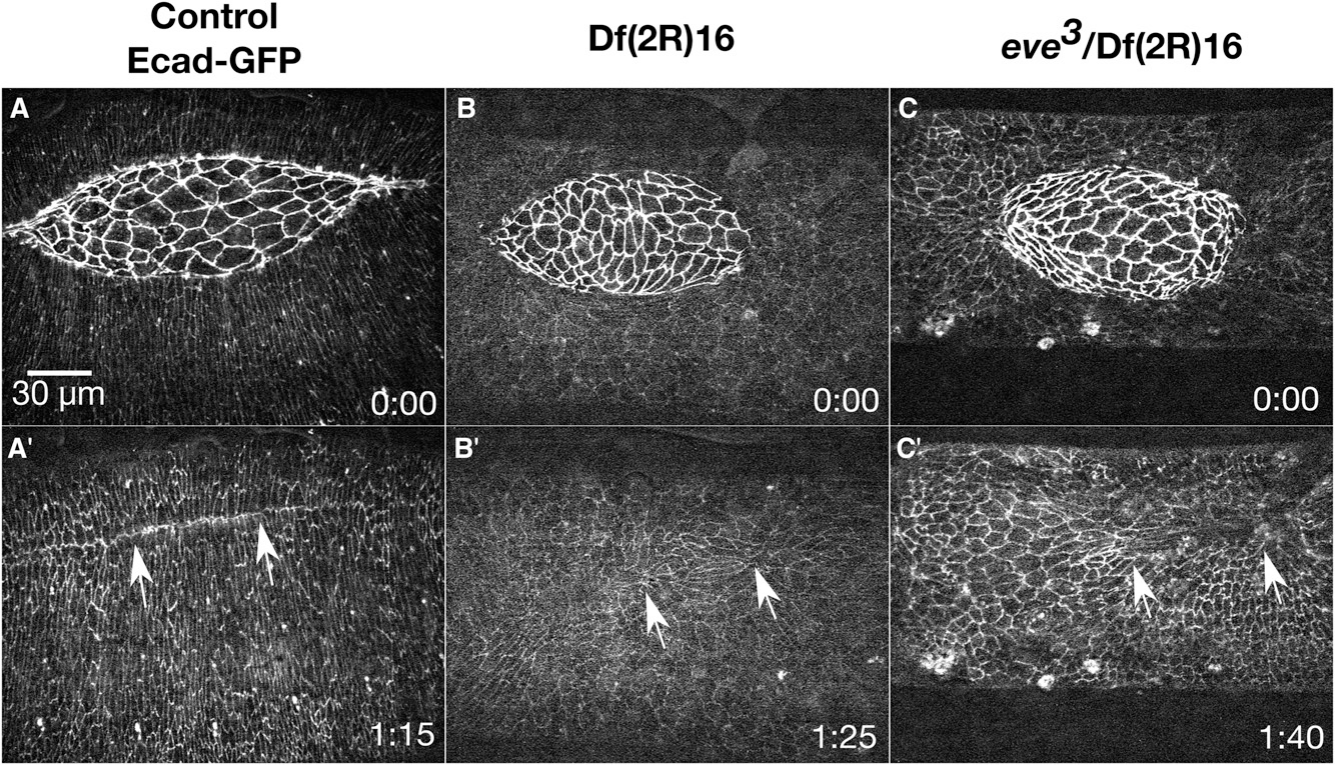
Deletion of *eve* is responsible for the phenotype of homozygous Df(2R)16 embryos. Time-lapse image series of similarly staged, Ecad-GFP labeled control (A-A’), homozygous Df(2R)16 (B-B’), and *eve^3^*/Df(2R)16 (C-C’) embryos. The top row shows embryos in mid-dorsal closure, and the second row shows the seam of the same embryos after closure completes, indicated by arrows. Anterior is to the left, posterior to right. Time is in hr:min. The scale bar in A applies to all micrographs (30 µm).

Throughout closure, embryos homozygous for Df(2R)16 have large, nearly isotropic lateral epidermal cells with the DME extended along the anterior-posterior axis (described above, Figure 4C-C’’’). In order to determine the gene responsible for this phenotype, we compared it to the phenotypes of two overlapping Dfs, Df(2R)15 and Df(2R)17 (Appendix A). Embryos homozygous for Df(2R)15 have no identifiable phenotype and embryos homozygous for Df(2R)17 have a more severe phenotype that fails in dorsal closure due to the known dorsal closure gene, *Jun-related antigen*. The phenotype for Df(2R)17 is so severe that we are unable to identify the shapes of the epidermal cells during dorsal closure, but they do appear abnormal at the onset of closure. We therefore imaged transheterozygous Df(2R)16/Df(2R)17 embryos and found that these embryos are indistinguishable from homozygous Df(2R)16 embryos (data not shown). Candidate genes deleted by the heteroallelic combination of Df(2R)16/Df(2R)17 include *even-skipped (eve)* as a possible gene of interest as it has a known germ-band extension defect and is involved in heart morphogenesis (Nüsslein-Volhard and Wieschaus 1980; Zallen and Wieschaus 2004; Buechling *et al.* 2009). Transheterozygous embryos for the null allele *eve^3^* and Df(2R)16 have large, isotropic lateral epidermal cells and are indistinguishable from homozygous Df(2R)16 embryos (Figure 8C-C’). Furthermore, we were able to completely rescue the dorsal closure phenotype of Df(2R)16 with a genomic *eve* rescue construct (Fujioka *et al.* 1995). We conclude that deletion of *eve* is responsible for the dorsal closure phenotype identified in Df(2R)16 embryos (manuscript in preparation, Fogerson *et al.*).

*eve* is a homeobox-containing transcriptional repressor that is required for establishing the even- and odd-parasegments of the early embryo through the repression of pair-rule and segment polarity genes (Nüsslein-Volhard and Wieschaus 1980; Macdonald *et al.* 1986; Biggin and Tjian 1989). In addition to patterning, *eve* also contributes to mesoderm and central nervous system development (Fujioka *et al.* 1995; Su *et al.* 1999). *eve*’s dorsal closure phenotype is likely due to changes in patterning and tissue specification prior to dorsal closure, as *eve* expression at the time of dorsal closure is localized to the neurons and anal pad (Frasch *et al.* 1987). We are currently following up on how disruption of the early patterning role of *eve* can lead to the observed changes in lateral epidermal cell morphology during dorsal closure.

### The deletion of three rows causes the Df(2R)60 phenotype and partially causes the Df(2R)61 phenotype

**Figure 9:**
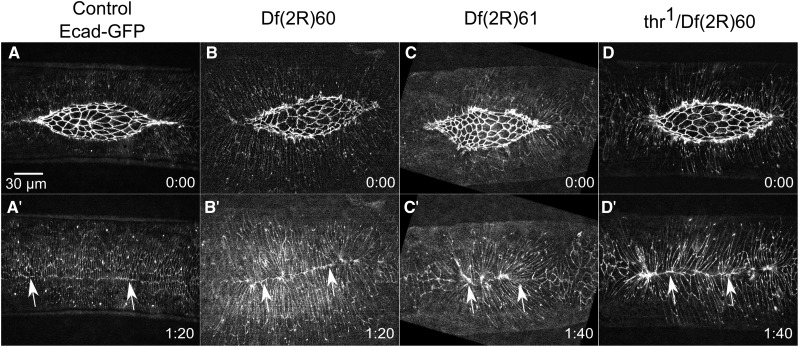
Deletion of *thr* is responsible for the phenotype of homozygous Df(2R)60 embryos and contributes to the phenotype observed in homozygous Df(2R)61 embryos. Time-lapse image series of similarly staged, Ecad-GFP labeled control (A-A’), homozygous Df(2R)60 (B-B’), homozygous Df(2R)61 (C-C’), and *thr^1^*/Df(2R)60 embryos (D-D’). The top row shows embryos in mid-dorsal closure, and the second row shows the seam of the same after closure completes, indicated by arrows. Anterior is to the left, posterior to right. Time is in hr:min. The scale bar in A applies to all micrographs (30 µm).

Embryos homozygous for either Df(2R)60 or Df(2R)61 have similar, fully penetrant phenotypes, although it is more severe in Df(2R)61. Embryos of both Dfs have very large lateral epidermal cells (larger than those found in *tum* mutants, compare Figure 9B’-D’ with Figure 7B’’, C’’) that stretch circumferentially along the dorsal-ventral axis similar to wild type embryos (compare Figure 9B and C with Figure 9A). The amnioserosa cells in both Dfs are similar to wild type, and embryos close at a normal rate. Embryos homozygous for either Df also have defects in zipping resulting in some slight scarring in the formed, dorsal epidermis of Df(2R)60 embryos and more severe scarring in the formed, dorsal epidermis of Df(2R)61 embryos (Figure 9B’ and C’). In addition, Df(2R)61 embryos have cell bunching in some DME cells, resulting in an oddly-shaped dorsal opening that likely contributes to the more severe scarring observed. The dorsal opening in Df(2R)60 embryos is eye-shaped, similar to wild type. The differences between the phenotypes in these Dfs suggest that Df(2R)61 deletes more than one gene affecting dorsal closure.

We imaged transheterozygous Df(2R)60/Df(2R)61 embryos and found that they have large lateral epidermal cells that are indistinguishable from homozygous Df(2R)60 embryos (data not shown). This indicates that the large cells seen in both Df(2R)60 and Df(2R)61 are due to the deletion of a gene in the overlapping genomic region. We identified *three rows* (*thr*) as a gene of interest in this region because mutations in *thr* result in mitosis and head involution defects (Liu *et al.* 1999; Pandey *et al.* 2005). Thr is part of the endoprotease separase complex that cleaves cohesin to permit sister chromatid separation (Jager *et al.* 2001). We imaged transheterozygous *thr^1^*/Df(2R)60 (*thr^1^* is a null allele; Philp *et al.* 1993) embryos in dorsal closure and found that they have large lateral epidermal cells similar to those observed in homozygous Df(2R)60 and Df(2R)61 embryos (Figure 9D and D’). In addition, the dorsal openings in *thr^1^*/Df(2R)60 embryos are eye-shaped and have slight scarring (but not the bunching that correlates with more severe scarring), in the formed dorsal epidermis, post-closure. Thus, the *thr^1^*/Df(2R)60 embryos are similar to homozygous Df(2R)60 embryos. We conclude that the deletion of *thr* causes the large lateral epidermal cells in Df(2R)60 and Df(2R)61 embryos and, moreover, large cells may lead to the observed slight scarring. We hypothesize that DME cell identity and differentiation is likely perturbed due to the failed cytokinesis prior to dorsal closure, thereby leading to the mis-match of segments from each lateral epidermal sheet during zipping (Perrimon and Desplan 1994; Mallo and Alonso 2013; Rousset *et al.* 2017). Furthermore, the additional gene or genes responsible for the cell bunching along the leading edge in Df(2R)61 embryos remains to be determined.

### Loss of jelly belly partially causes the phenotype in Df(2R)23 embryos

**Figure 10:**
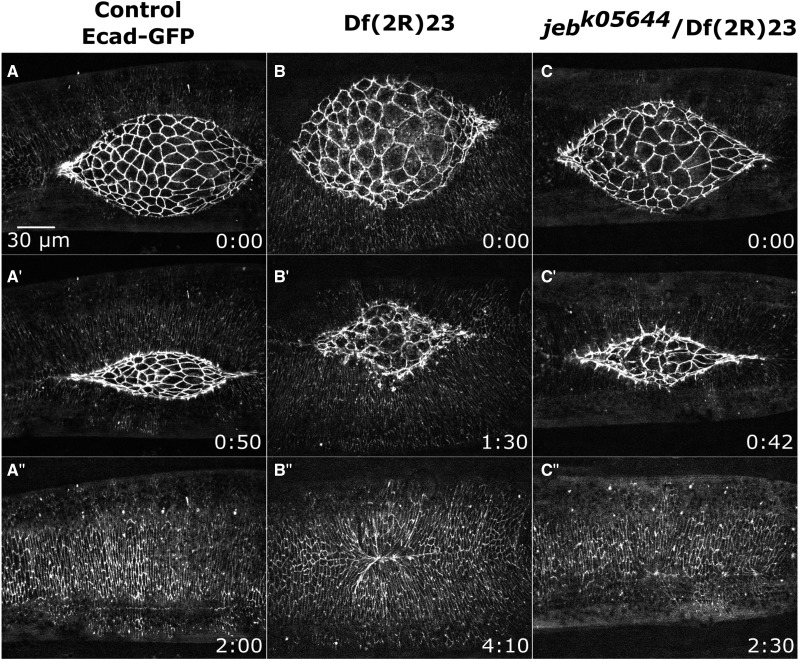
Deletion of jeb contributes to the Df(2R)23 phenotype. Time-lapse image series of similarly staged, Ecad-GFP labeled control (A-A’’), homozygous Df(2R)23 (B-B’’) and jeb^k05644^/Df(2R)23 embryos (C-C’’). Anterior is to the left, posterior to right. Time is in hr:min. The scale bar in A applies to all micrographs (30 µm).

Embryos homozygous for Df(2R)23 have a round dorsal opening and large amnioserosa cells that persist throughout closure (Figure 10B-B’ and described above, Figure 6C’). These embryos also have abnormal protrusions of E-cadherin fluorescence (cell-cell junctions) between the PAS cells in late closure that give the leading edge a jagged appearance (Figure 10B’). In addition, the yolk in Df(2R)23 embryos remains near the surface of the embryo throughout dorsal closure suggesting the amnioserosa may not have thickened properly. In wild type embryos, the yolk drops to a more interior position during closure (not shown). We identified *jelly belly* (*jeb*) as a candidate gene removed by Df(2R)23. Jeb is a signaling ligand for Alk which activates intracellular Ras/ERK and PI3K signaling pathways and is involved in a number of patterning and cellular signaling events in *Drosophila* development and regulation of intracellular vesicle transport respectively (Englund *et al.* 2003; Lee *et al.* 2003; Cheng *et al.* 2011). *jeb* is expressed in the somatic mesoderm where it is secreted and taken up by the visceral mesoderm (Weiss *et al.* 2001). In embryos mutant for *jeb*, the visceral mesoderm does not migrate as in wild type and does not differentiate, thereby disrupting the formation of the midgut (Campos-Ortega and Hartenstein 1997; Weiss *et al.* 2001; Lee *et al.* 2003).

We imaged transheterozygous *jeb^k05644^*/Df(2R)23 embryos in dorsal closure and found that they have a weaker phenotype than that observed for homozygous Df(2R)23 embryos (compare Figure 10C to B). The yolk in *jeb^k05644^*/Df(2R)23 embryos remains just below the surface of the amnioserosa, and sometimes bulges out at the end of closure similar to Df(2R)23 embryos (not shown). In addition, protrusions of the E-cadherin belts under the lateral epidermal cells gives the appearance of a jagged leading edge, similar to that seen in Df(2R)23 embryos (compare Figure 10C’ to 10B’). The dorsal opening is slightly round in some *jeb^k05644^*/Df(2R)23 embryos, but this is not as severe as in Df(2R)23 embryos. We conclude that the deletion of *jeb* is partially responsible for the phenotype observed in Df(2R)23 embryos, but that the deletion of one or more other genes contributes to the Df phenotype, specifically the round dorsal opening.

### Deficiency with a 2^nd^ site mutation

**Figure 11:**
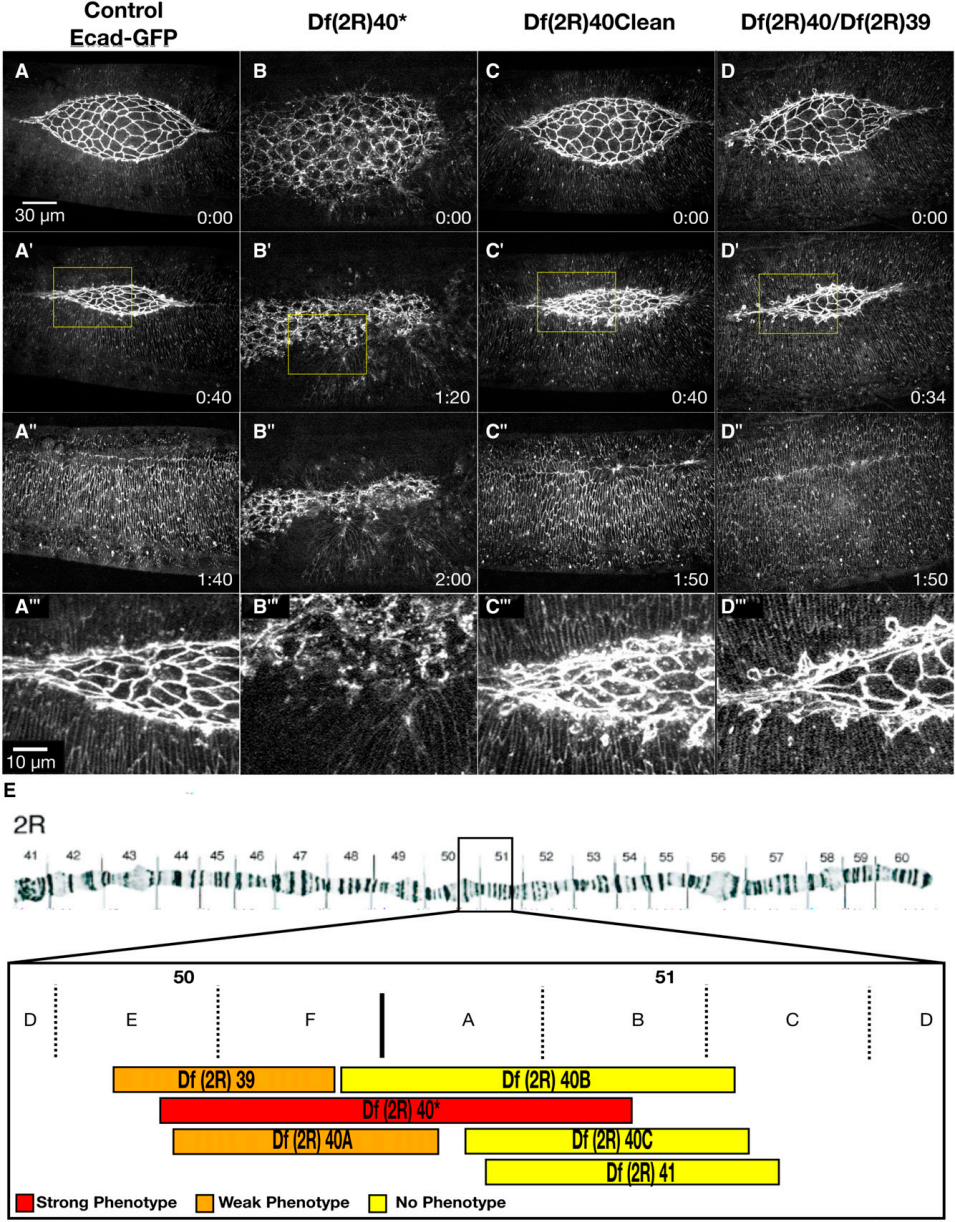
The severe phenotype in embryos homozygous for Df(2R)40 is from a lesion outside the genomic interval deleted by this Df. Time-lapse image series of Ecad-GFP labeled control (A-A’’’), homozygous Df(2R)40* (B-B’’’), homozygous Df(2R)40Clean (C-C’’’), and Df(2R)40/Df(2R)39 embryos (D-D’’’). A’’’, B’’’, C’’’, and D’’’ show magnified views of the yellow boxed areas of the amnioserosa in the corresponding panels. Df(2R)40* (asterisk indicates that the Df(2R)40 has a severe dorsal closure phenotype because of a lesion that falls outside of the mapped Df, see text) and Df(2R)40Clean is outcrossed to w^1118^. A cytological map schematic of the right arm of chromosome 2 demonstrates the region removed in Df(2R)40 and overlapping Dfs (E). The image of the polytene chromosome was previously published in (Halsell and Kiehart 1998). Homozygous embryos of Df(2R)40 have a strong dorsal closure phenotype (denoted in red). Transheterozygous embryos of Df(2R)40 with Df(2R)39 or Df(2R)40A have a weak dorsal closure phenotype (denoted in orange). Transheterozygous embryos with Df(2R)40* with Df(2R)40B, Df(2R)40C, or Df(2R)41 have no dorsal closure phenotype (denoted in yellow). Thus, the lesion responsible for Df(2R)40*’s severe phenotype must be outside the defined breakpoints of Df(2R)40, since no Df spanning this region is as severe as Df(2R)40*, and the outcrossed Df(2R)40 (referred to as Df(2R)40Clean) does not phenocopy the severe phenotype. Anterior is to the left, posterior to right. Time is in hr:min. The scale bar in A applies to panels A-D’’ (30 µm). Scale bar in A’’’ applies to panels A’’’-D’’’ (10µm).

Embryos homozygous for Df(2R)40* have a severe dorsal closure phenotype including disorganized lateral epidermal cells and amnioserosa cells. There is also tearing between the DME and PAS cells (Figure 11B-B’’’). We have found that this phenotype is due to a lesion outside of the mapped Df (which is why we mark this DC Df with an asterisk). We attempted to narrow the region of the genome responsible for this phenotype by crossing Df(2R)40* to five overlapping Dfs which collectively delete the entire region removed by Df(2R)40 (Figure 11E, Appendix B, Gramates *et al.* 2017). Contrary to homozygous Df(2R)40* embryos, embryos transheterozygous for Df(2R)40* with Df(2R)39 and Df(2R)40A have normally organized lateral epidermis and amnioserosa cells which remain intact through the completion of closure (Figure 11D-D’’’ and data not shown). In mid- to late-closure, the PAS cells in these embryos protrude out under the lateral epidermal cells giving the impression that the dorsal opening has a jagged periphery (Figure 11D’ and D’’’). These embryos complete closure with little or no scarring in the formed, dorsal epithelium (Figure 11D’’). Embryos transheterozygous for Df(2R)40* and the three remaining overlapping Dfs (Df(2R)40B, Df(2R)40C and Df(2R)41) have no identifiable dorsal closure phenotype (data not shown). We concluded that the deletion of a gene or genes in the overlapping genomic region of Df(2R)39 and Df(2R)40A causes the weak PAS protrusion phenotype but none of the overlapping Dfs result in the severe phenotype identified in homozygous Df(2R)40* (Figure 11E). Thus, the severe phenotype of Df(2R)40* could be due to a lesion on the chromosome outside of the defined Df deletion or it could be an additive effect of multiple genes removed, not all of which are deleted by any one of the overlapping Dfs. To investigate the former, we outcrossed Df(2R)40* flies to a wild type stock and found the Df(2R)40*Clean stock recapitulated the less severe phenotype of the Df(2R)40*/Df(2R)39 transheterozygous embryos, thus the severe phenotype identified in Df(2R)40* is due to a lesion outside the deficiency region. This result demonstrates the importance of verifying a phenotype by crossing independently derived alleles or by crossing an allele to a Df.

### Df(2R)36 does not delete the expected region

Df(2R)36 is one of the largest Dfs in the 2R Df kit and its genomic deletion overlaps all of Df(2R)37 and Df(2R)38 and parts of Df(2R)35, 39 and 40 (Gramates *et al.* 2017). Df(2R)36 surprisingly shows no phenotype, whereas Df(2R)37 has slow zipping due to the removal of *shot* (Takács *et al.* 2017) and large lateral epidermal cells due to the deletion of *tum* (Figure 7C’). The lack of a phenotype in Df(2R)36 could result from (1) the deleted region is not, after all, deleted,(2) a duplication event copied this interval somewhere else on the chromosome, or (3) the loss of other genes in Df(2R)36 outside of Df(2R)37 can suppress the Df(2R)37 phenotypes. To test these possibilities, we crossed Df(2R)36 and Df(2R)37 to the known lethals *shot^SF20^* and *tum^AR2^*, to Df(2R)38, and to each other. All alleles are balanced with a balancer containing *Duox^Cy^*, a dominant allele that causes curled wings. We then determined complementation of lethality to viability through the presence of straight-winged progeny. Df(2R)36/37, Df(2R)36/38, Df(2R)36/shot^SF20^ and Df(2R)36/tum^AR2^ are all viable whereas Df(2R)37/*shot^SF20^* and Df(2R)37/*tum^AR2^* are lethal. This suggests that the expected deletion region of Df(2R)36, which should include all of Df(2R)37 and Df(2R)38 is NOT completely deleted or there is a duplication of this region somewhere else on the chromosome.

### E-cadherin overexpression can rescue phenotypes

In this Df screen, we utilized Ecad-GFP to label the cell junctions that maintain the mechanical integrity of the amnioserosa, the lateral epidermis and the interface between the two tissues. Fluorescent imaging of Ecad-GFP allows facile identification of the cell shapes at the level of the adherens junctions in both tissues and helps to identify irregularities in the kinematics of cell shape changes that contribute to morphogenesis. Ecad-GFP is a ubiquitously driven, fully functional, GFP labeled E-cadherin that was shown to be able to rescue null alleles of the E-cadherin-encoding gene *shotgun* (*shg*; Oda and Tsukita 2001). Because of its ability to rescue the deletion of *shg*, we hypothesized that Ecad-GFP might also rescue phenotypes that are caused by deletion of *shg* or by the deletion of genes that interact with *shg*.

### Ubiquitously expressed E-cadherin-GFP can rescue shotgun (E-cadherin) deletion

**Figure 12:**
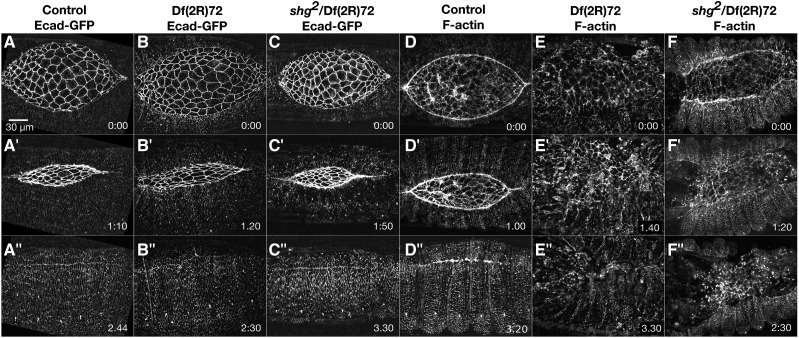
Ubiquitously expressed Ecadherin-GFP rescues the deletion of *shotgun* (*shg*). Time-lapse image series of Ecad-GFP labeled control (A-A”), homozygous Df(2R)72 (B-B”), and *shg^2^*/Df(2R)72 embryos (C-C”). Time-lapse image series of F-actin (sGMCA) labeled control (D-D”), homozygous Df(2R)72 (E-E”), and *shg^2^*/Df(2R)72 embryos (F-F”). *shg^2^*/Df(2R)72 embryos labeled with Ecad-GFP have no dorsal closure phenotype, while s*hg^2^*/Df(2R)72 embryos labeled with sGMCA have a very severe phenotype. Anterior is to the left, posterior to the right. Time is in hr:min. The scale bar in A applies to all micrographs (30 µm).

**Figure 13:**
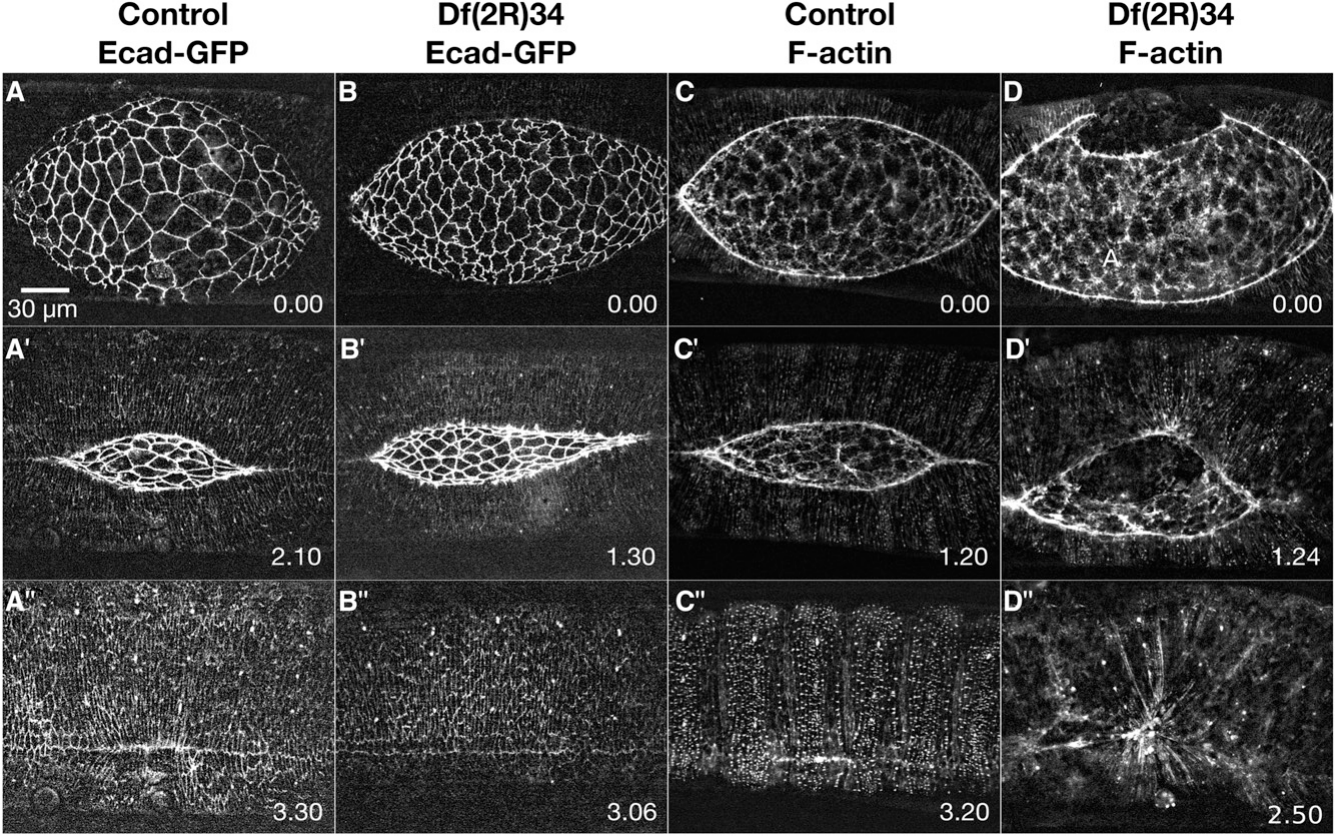
Df(2R)34 dorsal closure defects are rescued by overexpression of E-cadherin. Time-lapse image series of Ecad-GFP labeled control embryos (A-A’’) and homozygous Df(2R)34 embryos (B-B’’). F-actin (sGMCA) labeled control embryos (C-C’’) and homozygous Df(2R)34 embryos (D-D’’). Embryos homozygous for Df(2R)34 and labeled with Ecad-GFP have a very weak dorsal closure phenotype; however, when embryos homozygous for Df(2R)34 are labeled with sGMCA, the dorsal closure phenotype is much more severe. Anterior is to the left, posterior to right. Time is in hr:min. The scale bar in A applies to all micrographs (30 µm).

To test the ability of Ecad-GFP to rescue the deletion of *shg*, we investigated Df(2R)72, the 2R Df that deletes *shg*. As shown above, embryos homozygous for Df(2R)72 labeled with Ecad-GFP fail to form the anterior canthus (5 of 6 embryos imaged, Figure 5D-D’’’ and Figure 12B-B’’). One embryo forms an anterior canthus, but zipping is inhibited at that canthus (data not shown). We confirmed that Ecad-GFP can rescue severe (including null) alleles of *shg* by imaging Ecad-GFP-expressing embryos of the genotype *shg^2^*/Df(2R)72. Dorsal closure in all six such embryos imaged is indistinguishable from control Ecad-GFP embryos (Figure 13C-C’’). Indeed, ubiquitously expressed Ecad-GFP is able to rescue *shg^2^*/Df(2R)72 animals through embryogenesis and even to adulthood.

It is possible that our version of Df(2R)72 was improperly labeled and does not delete the appropriate region of the genome and therefore does not delete *shg*. To test this, we imaged homozygous Df(2R)72 with sGMCA, a fluorescence imaging marker that labels F-actin (Kiehart *et al.* 2000). We hypothesize that if Df(2R)72 removes *shg*, its homozyogous phenotype should be more severe in an imaging background that does not include Ecad-GFP. In fact, F-actin labeled embryos homozygous for Df(2R)72 have a much more severe phenotype than those labeled with Ecad-GFP (compare Figure 12E-E’’ with Figure 12B-B’’) and are also more severe than previously published *shg* alleles, further indicating that Df(2R)72 deletes a gene or genes other than *shg* that contributes to closure (Gorfinkiel and Martinez-Arias 2007). Very few of these embryos make it to dorsal closure, and in those that do, the amnioserosa and lateral epidermis are difficult to identify. They are severely disorganized, and they lack increased F-actin at the purse strings (Figure 12E-E’). Although it appears that closure in these embryos continues, the tissue is severely puckered, and it is not clear if closure completes (Figure 12E-E’’). In addition, we imaged F-actin labeled embryos of the genotype *shg^2^*/Df(2R)72 and these also are more severe than *shg^2^*/Df(2R)72 embryos labeled with Ecad-GFP (compare Figure 12F-F’’ with Figure 12C-C’’). This confirms that Ecad-GFP is able to rescue the deletion of *shg* and that another gene or genes deleted by Df(2R)72 is responsible for anterior canthus formation.

### E-cadherin overexpression rescues Df(2R)34

To test the possibility that ubiquitously expressed E-cadherin-GFP is rescuing or ameliorating any other Df phenotypes, we imaged F-actin labeled embryos homozygous for six randomly chosen Dfs that cause weak phenotypes when labeled with Ecad-GFP. Of the six Dfs imaged in an F-actin labeled background, five cause a similar phenotype to that seen in an Ecad-GFP background. In contrast, one Df, Df(2R)34, has a more severe phenotype when imaged in other backgrounds compared to Ecad-GFP (compare Figure 13D-D’’ with Figure 13B-B’’).

We further evaluated the dorsal closure phenotype in Df(2R)34 homozygous embryos. In the course of the screen, we had imaged six Ecad-GFP labeled Df(2R)34 embryos. Three of these embryos have a slight cigar-shaped dorsal opening, so slight that a similar, weak, cigar-shaped phenotype is sometimes observed in control embryos (compare Figure 13B’ with Figure 13A’). Because of the low penetrance of this weak phenotype, which overlaps with control embryos, this Df was classified as having a weak phenotype and was set aside. However, we imaged 10 Df(2R)34 embryos in an F-actin labeled sGMCA background as described above. We found 8 of 10 embryos develop holes or tearing along the amnioserosa/lateral epidermis border (Figure 13D-D’). These holes form at any time from early closure to late closure and persist throughout closure. This suggests that Df(2R)34 deletes a gene that contributes to adhesion between the DME cells and the PAS cells, an activity which is partially rescued by the expression of Ecad-GFP. Remarkably, closure completes in all embryos with severe scarring (Figure 13D’’).

To test the possibility that the observed phenotype is a result of an interaction between one or more of the genes deleted by Df(2R)34 and expression of sGMCA, we also imaged Df(2R)34 in the microtubule-labeled GFP background, Jupiter-GFP. Of these, 4 of 6 embryos have holes or tearing along the amnioserosa/lateral epidermis border and all six embryos have scarring (data not shown). We conclude that neither sGMCA nor Jupiter-GFP causes the phenotype and that a gene deleted in Df(2R)34 prevents tearing at the amnioserosa/lateral epidermis border. This activity is rescued by the expression of the Ecad-GFP in the presence of endogenously expressed E-cadherin, indicating that over-expression of cadherin can rescue the dorsal closure defects that characterize Df(2R)34 and establishing a genetic interaction between one or more genes deleted by Df(2R)34 and *shg*. This observation further indicates that there may be other Dfs in our screen that show a weak or no phenotype when imaged with Ecad-GFP because of rescue by the expression of Ecad-GFP.

## Conclusions

**Figure 14:**
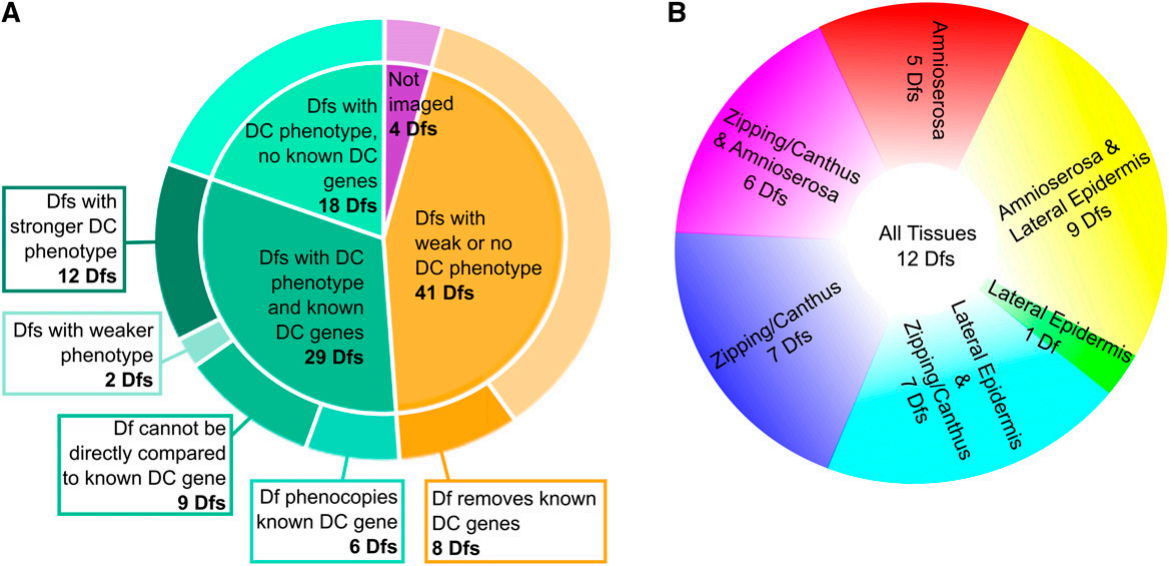
Pie chart summaries indicate the number of Dfs that cause DC phenotypes and the tissues they affect. Of the 92 Dfs in the 2R Df kit, 47 cause DC phenotypes and 18 of these do not delete a known DC gene. Some of the Dfs have different DC phenotypes than the DC gene they delete, suggesting that there may be additional, new DC genes in the deleted interval (A). The 47 DC Dfs cause phenotypes in one or more tissues or structures. Note that the color coding in Appendix A corresponds to the categories in this pie chart (B).

Through live imaging of homozygous Df embryos from the Bloomington 2R Df kit, we identified 47 of 92 Dfs which cause a phenotype in dorsal closure, so called dorsal closure Dfs (Figure 14A-B). We grouped Dfs by the severity of their recessive, dorsal closure phenotypes and according to the tissues they affect. The dorsal closure Dfs delete dorsal closure genes that contribute to the function of most if not all, tissues, cell types, and processes that contribute to dorsal closure. These include the amnioserosa, the lateral epidermis, zipping/canthus formation and the interface between the DME and PAS cells, presumably due to defects in the function of DME cells, PAS cells or both (Figure 14B).

We were surprised that all eighty-eight Dfs we imaged made it to the dorsal closure stage, as we expected that some would remove genes necessary for earlier processes and would lead to the failure of embryogenesis. Indeed Df(2R)72, removing *shg*, which encodes E-cadherin, may not make it to closure in the absence of Ecad-GFP. We did identify eleven Dfs that are irregular at the start of closure (*e.g.*, irregular cell shapes or sizes and incomplete germ band retraction) which indicates disruption of earlier processes (see Appendix A). We surmise that even such Dfs have the potential of providing insight into the mechanisms of closure. How can dorsal closure complete when it begins with compromised tissues? Our live imaging approach allows us to dissect the robust nature of this morphogenetic process. Although some Dfs begin closure with defects, some also get significantly more severe as closure proceeds. This could be due to the effect of the force-producing dorsal closure process on already compromised tissues, or it could reflect genes in the Df region that also have a function during dorsal closure. For example, the known dorsal closure gene *Egfr* has a pre-dorsal closure defect and is removed by Df(2R)75, a Df that, as would be expected, also has a pre-dorsal closure defect (Shen *et al.* 2013).

Our screen would miss key genes whose products are required for closure but are loaded maternally and perdure through dorsal closure stages. Such genes, although required for closure, would not need to be zygotically transcribed. Because none of the Dfs in the Bloomington Df kit are homozygous viable, extending the Df screen to identify maternal effect genes is not feasible.

Thus far, this screen has helped us to identify four novel genes that affect closure when deleted and we are continuing to use overlapping Dfs/duplications, existing null alleles, and CRISPR to make additional null alleles of individual genes and groups of genes. In the process of trying to narrow down the genomic region of interest for one Df, by crossing it to overlapping Dfs, we discovered that one lesion that caused a severe dorsal closure phenotype is elsewhere on the chromosome, outside the expected deletion of the Df. This serves as an important reminder that alleles should be tested against an independently generated allele to verify that the identified phenotype is due to disruption of the region of interest.

Previous to this Df screen, ∼140 genes had been identified as affecting dorsal closure (*i.e.*, “dorsal closure genes”). Of the 47 Dfs that we found to cause a dorsal closure phenotype, 18 remove no known dorsal closure genes (Figure 14A). We anticipate that each of these will lead to the identification of one or more novel dorsal closure genes. In addition, 12 Dfs that remove known dorsal closure genes, cause a stronger phenotype than that observed for the null alleles of the dorsal closure gene(s) they remove (see Table 5 and Appendix A). This suggests that these Dfs also remove one or more additional and novel dorsal closure genes. Therefore, we anticipate that 2R has a minimum of ∼30 novel dorsal closure genes that will be identified from this screen. This minimum does not include the possibility of some Dfs removing more than one dorsal closure gene and indeed, we have already identified such Dfs. This pilot screen suggests that with the continuation of this Df screen to the remaining chromosomal arms, we would expect to identify ∼165 or more novel dorsal closure genes, more than doubling the number of genes currently identified to be involved in the discrete processes that comprise closure. The dorsal closure Df and mutant gene phenotypes we have documented demonstrate that successful closure requires the interaction of multiple cells, cell types and a number of distinct biological processes. These new dorsal closure genes will be a valuable asset to help our understanding of the molecular mechanisms that drive the discrete processes that orchestrate closure, a conceptually simple, yet biologically complex morphogenic movement. In addition, these phenotypes will help us to better understand the many unanswered questions about the mechanics of dorsal closure and more broadly the biology of cell sheet morphogenesis throughout phylogeny.
